# *Lactobacillus plantarum *gene clusters encoding putative cell-surface protein complexes for carbohydrate utilization are conserved in specific gram-positive bacteria

**DOI:** 10.1186/1471-2164-7-126

**Published:** 2006-05-24

**Authors:** Roland Siezen, Jos Boekhorst, Lidia Muscariello, Douwe Molenaar, Bernadet Renckens, Michiel Kleerebezem

**Affiliations:** 1 Wageningen Centre for Food Sciences (WCFS), Wageningen, The Netherlands; 2 Centre for Molecular and Biomolecular Informatics (CMBI), Radboud University Nijmegen, The Netherlands; 3 NIZO food research, Ede, The Netherlands

## Abstract

**Background:**

Genomes of gram-positive bacteria encode many putative cell-surface proteins, of which the majority has no known function. From the rapidly increasing number of available genome sequences it has become apparent that many cell-surface proteins are conserved, and frequently encoded in gene clusters or operons, suggesting common functions, and interactions of multiple components.

**Results:**

A novel gene cluster encoding exclusively cell-surface proteins was identified, which is conserved in a subgroup of gram-positive bacteria. Each gene cluster generally has one copy of four new gene families called *cscA, cscB, cscC *and *cscD*. Clusters encoding these cell-surface proteins were found only in complete genomes of *Lactobacillus plantarum*, *Lactobacillus sakei*, *Enterococcus faecalis*, *Listeria innocua*, *Listeria monocytogenes*, *Lactococcus lactis ssp lactis *and *Bacillus cereus *and in incomplete genomes of *L. lactis ssp cremoris*, *Lactobacillus casei*, *Enterococcus faecium*, *Pediococcus pentosaceus*, *Lactobacillius brevis*, *Oenococcus oeni*, *Leuconostoc mesenteroides*, and *Bacillus thuringiensis*. These genes are neither present in the genomes of streptococci, staphylococci and clostridia, nor in the *Lactobacillus acidophilus *group, suggesting a niche-specific distribution, possibly relating to association with plants. All encoded proteins have a signal peptide for secretion by the Sec-dependent pathway, while some have cell-surface anchors, novel WxL domains, and putative domains for sugar binding and degradation. Transcriptome analysis in *L. plantarum *shows that the *cscA-D *genes are co-expressed, supporting their operon organization. Many gene clusters are significantly up-regulated in a glucose-grown, *ccpA-*mutant derivative of *L. plantarum*, suggesting catabolite control. This is supported by the presence of predicted CRE-sites upstream or inside the up-regulated *cscA-D *gene clusters.

**Conclusion:**

We propose that the CscA, CscB, CscC and CscD proteins form cell-surface protein complexes and play a role in carbon source acquisition. Primary occurrence in plant-associated gram-positive bacteria suggests a possible role in degradation and utilization of plant oligo- or poly-saccharides.

## Background

Most Gram-positive bacteria are known to produce a multiplicity of extracellular proteins, many of which are destined to become attached to the cell surface [1-5]. These surface-exposed proteins serve to communicate and interact with the environment. Particularly in pathogenic streptococci, staphylococci and *Listeria*, they are often of primary importance in bacterial adhesion, invasion and interaction with host cells [6-8]. Cell-surface proteins are also known to play an essential role in providing nutrition to the cell through binding, degradation and uptake of carbon and nitrogen substrates. Many cell-surface proteins have a multi-domain architecture, and share various structural features including secretion signal peptides, cell-anchoring domains or motifs, cell-wall spanning regions, and repeated domains of various functions. In some cases, multiple proteins join forces to form large extracellular complexes that provide both binding and enzymatic functionalities, such as the cellulosomes of anaerobic bacteria (e.g. *Clostridium, Ruminococcus*) for degradation of and growth on cellulose, the main structural component of plant cell walls [9-13].

Even though the function of a variety of extracellular proteins of Gram-positive bacteria has been characterized experimentally, recent genome sequencing efforts have led to the prediction of hundreds of encoded extracellular proteins of unknown function. Many of these appear to belong to conserved homologous families of hypothetical extracellular proteins, suggesting common functions in different bacterial species. While it is often possible to detect known cell-anchoring domains in these proteins, such as (i) amino- or carboxy-terminal membrane-spanning anchors, (ii) peptidoglycan anchors covalently bound through their LPxTG motif [4,14-18], (iii) amino-terminal lipid-bound anchors [19], and (iv) a variety of domains binding non-covalently to peptidoglycan, teichoic acids [20] or surface polysaccharides, the main function(s) of these encoded cell-surface proteins in their interaction with the environment remains elusive.

*Lactobacillus plantarum *is a gram-positive bacterium that is encountered in many different environmental niches, as it is associated with various plants [21-24], it occurs in several food and feed fermentations [25-28], and it is a natural inhabitant of the gastrointestinal tract of humans and animals [29,30]. Analysis of the 3.3 Mbp genome sequence of *L. plantarum *WCFS1 revealed over 200 putative extracellular proteins based on the presence of an N-terminal signal peptide [31]. The vast majority of these proteins contained at least one of the cell-anchoring motifs described above. A new C-terminal domain designated WxL was found in 19 proteins of *L. plantarum*. More recently, fifteen proteins with a WxL-like domain were identified in the genome of *Lactobacillus sakei *23 K [32], and found to be encoded in gene clusters that potentially encode a multicomponent complex of unknown function on the bacterial surface. In search of putative functions for the encoded hypothetical extracellular proteins, and their possible relation to niche adaptation, we have now discovered that 35 of the cell-surface proteins of *L. plantarum *are encoded in nine paralogous gene clusters. Four different types of novel protein families are represented in these gene clusters. We present bioinformatics and experimental evidence that the encoded proteins are functionally coupled and possibly form a cell-surface protein complex that could play a role in sugar metabolism. A genome-wide search revealed similar gene clusters in a specific subgroup of mainly plant-associated Gram-positive bacteria, and we therefore postulate a role in degradation of (complex) plant polysaccharides.

## Results

### Cell-surface clusters in Lactobacillus plantarum WCFS1

**Figure 1:**
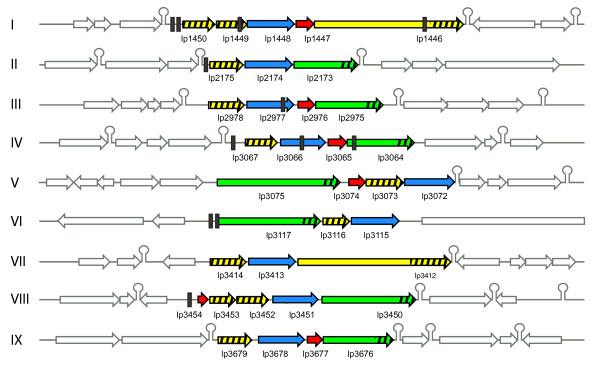
***Csc *gene clusters found in *Lactobacillus plantarum *WCFS1**. Genes are color-coded according to family: *cscA *(blue), *cscB *(yellow), *cscC *(green), *cscD *(red); other genes are not coloured. Positions of encoded WxL1 domains (in CscB) and WxL2 domains (in CscC) are striped. Predicted CRE sites are indicated by black vertical bars (see also Table 3). Predicted terminators are indicated by loop symbols.

**Figure 2:**
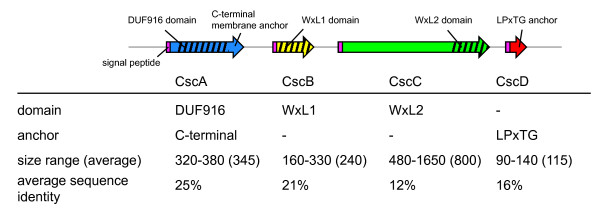
**Schematic summary of the characteristics of Csc families**. The summary is based on all proteins found in the genomes listed in Table 1. The size range refers to the entire proteins. Approximate position and size of domains (DUF916, WxL1, WxL2), identified by Hidden Markov models, are indicated by stripes. The average sequence identity refers to the entire proteins, and is particularly low for the CscC proteins, which only have WxL2 domains in common, and for CscD proteins, which only have the LPxTG anchors in common.

Analysis of the chromosome indicated that many of the predicted extracellular proteins are encoded in clusters of 3–6 genes [31]. A closer inspection reveals that nine clusters encode proteins which can be divided into 4 different classes or families based on amino acid sequence similarity, domain and motif characteristics (Table 1; Fig. 1; see details in additional files 1, 2). All of the 35 encoded Csc proteins (cell-surface complex) have normal signal peptides for secretion via the Sec-dependent pathway [33] and processing by the signal peptidase I. Most of the Csc proteins and their domains are of unknown function since they do not have significant similarity to proteins of known function (see below for details). The four families can be easily distinguished based on domain composition. The CscA proteins are all predicted to contain a conserved domain of unknown function (PFAM: DUF916) as well as a C-terminal transmembrane anchor. CscB and CscC proteins are characterized by a novel domain of 160–190 residues, which we have termed WxL since it contains two characteristic conserved sequence motifs containing the WxL signature (Fig. 1)[31]. The CscB proteins are on average 240 amino acids in size and consist almost entirely of the WxL domain, while the CscC proteins are much larger with an average size of 800 amino acids and have a variable N-terminus. Since the WxL domains of the CscB and CscC proteins can be distinguished based on sequence characteristics such as the distance between the conserved WxL residues, they were considered as two different families (WxL1 for the CscB proteins, WxL2 for the CscC proteins). Finally, members of the CscD family all have a C-terminal LPxTG-type motif for sortase-mediated covalent anchoring to the peptidoglycan layer [4,14], and are uncharacteristically small for LPxTG-anchored proteins. Figure 2 summarizes the characteristics of the four Csc family members. The individual families will be discussed in more detail below.

**Table 1:** Occurrence of cell-surface clusters and genes in genomes

		**genes**^#^			
		
**Species**	**clusters**	***cscA***	***cscB***	***cscC***	***cscD***
**Complete genome sequences**					
*Lactobacillus plantarum *WCFS1	9	9	13	8	6
*Lactobacillus sakei *23K	8	10*	8	6*	2
*Enterococcus faecalis *V583	6	7	17	4	5
*Lactococcus lactis *IL1403	3	4 *	6	2	3
*Listeria innocua *Clip11262	3	4	3	3	2
*Listeria monocytogenes *EGD-e	2	2	2	2	1
*Bacillus cereus *ZK	1	1	2	1	1
*Bacillus anthracis *A2012 *(plasmid)*	1	1*	1	1	0
**Incomplete genome sequences**					
*Lactococcus cremoris *SK11	5	5 *	7	3	3
*Lactobacillus casei *ATCC367	4	5*	5*	3*	3
*Enterococcus faecium *DO	3	4	3 *	3	1
*Pediococcus pentosaceus ATCC25745*	2	2	2	1	1
*Leuconostoc mesenteroides ATCC8293*	1	1	1	1	1
*Oenococcus oeni *PSU-1	1	1 *	1	0	0
*Bacillus thuringiensis *ATCC35646	1	1	2	1	1
*Lactobacillus brevis *ATCC367	?	0	1	6	0
					
**Total**	50	57	74	45	30

^# ^Seven of these *csc *genes are found outside the gene clusters in complete genomes (details in additional file 1).

* Some genes contain frame shifts, stop codons or truncations (details in additional file 2).

### Cell-surface clusters in other bacteria

The NCBI and ERGO genome databases were searched for the presence of Csc family members and *csc*-like gene clusters. Clusters encoding these cell-surface proteins were found in the complete genomes of *Lactobacillus plantarum *(9 clusters)[31], *Lactobacillus sakei *(8)[32], *Enterococcus faecalis *(6), *Listeria innocua *(3), *Listeria monocytogenes *(2), *Lactococcus lactis ssp lactis *(3), *Bacillus cereus *ZK (1), *Bacillus cereus *10987 (1, on plasmid) and *Bacillus anthracis *(1, on plasmid) (Table 1). The *csc *clusters were also found in the incomplete genomes of *L. lactis ssp cremoris *(5 clusters, of which one cluster on a plasmid), *Lactobacillus casei *(3), *Enterococcus faecium *(3), *Pediococcus pentocaseus *(2), *Oenococcus oeni *(1), *Leuconostoc mesenteroides *(1), and *Bacillus thuringiensis *(1). Details of all *csc *gene clusters and encoded proteins can be found in additional files 1, 2, 3. In several cases *csc *genes are still unidentified in incomplete genomes because the clusters are on small contigs. Each gene cluster generally has one copy each of the 4 new gene families *cscA, cscB, cscC *and *cscD*, although some variation is observed. A single copy of the *cscA *is always present, while 1–4 different *cscB *genes occur in the gene clusters. Although single *cscC *and *cscD *genes are usually present, they are missing in a few clusters. All encoded proteins have a regular signal peptide for secretion by the Sec-dependent pathway.

### Evidence of gene clusters as functional units

There are many indications that these gene clusters are functional units, i.e. that the genes are transcribed coordinately, and that the encoded gene products function together in a pathway or protein complex.

C*sc *genes are nearly exclusively found in these gene clusters, with very few exceptions outside the clusters. The clusters rarely contain other genes than the *csc *family members, as based on the criteria of correct gene orientation, small intergenic distance and absence of predicted termination sequences. In all *csc *clusters, the genes are oriented in the same transcriptional direction and usually have intergenic regions smaller than 100 nucleotides, suggestive of an operon structure. In general, the *csc *gene clusters are bounded by terminators on both sides (Fig. 1). One complete gene cluster (LLX-I) on the *L. lactis ssp lactis *IL1403 chromosome is exactly bordered by IS981 elements, and several other clusters are flanked on one side by IS elements, suggesting that some of these gene clusters have been transferred as a unit. Moreover, complete *csc *gene clusters are found on plasmids of *L. lactis *SK11 [34], *B. anthracis *and *B. cereus *(see additional file 1), suggesting that these genes can be transferred between strains or species via these mobile genetic elements.

Comparative DNA microarray-based genotyping analysis of 20 strains of *Lactobacillus plantarum *revealed considerable variation in the presence/absence of different DNA regions in individual strains as compared to strain WCFS1 [35]. In general, the *csc *clusters of *L. plantarum *WCFS1 appear to be highly conserved in other strains. However, the entire cluster LPL-IX (LPL3676-3679) appears to be missing in 3 of the 20 strains analyzed, while the genes flanking this cluster appear to be present. Again, this suggests that the entire cluster can be excised or inserted as a functional unit.

### Domain and function prediction of Csc proteins

#### CscA family

The CscA proteins are found to belong to the PF06030 Pfam family (or DUF916, bacterial proteins of unknown function). In addition to the N-terminal signal peptide, these proteins all contain a predicted C-terminal trans-membrane helix, which presumably serves to anchor them in the cell membrane (see full sequence alignment in additional file 6). Each *csc *gene cluster generally encodes only a single CscA protein (see additional file 1). The CscA-family members are fairly uniform in size (320–380 residues), and the large majority are predicted to be very basic proteins with a pI above 9.0 (see additional file 2).

#### CscB family

The CscB family members are also fairly uniformly sized (190–280 residues, with a few exceptions), and typically have an acidic pI of 4–5. These proteins are not yet described in the Pfam or COG databases. We have defined the C-terminal domain of about 160–190 residues as the "WxL1" domain (Fig. 1; see full sequence alignment in additional file 7) since it contains two highly conserved sequence motifs Trp-x-Leu. Preceding the first Trp-x-Leu motif is a highly conserved Asp-x-Arg-Gly sequence. Most family members have a short Pro-rich region between the signal peptide and the WxL1-domain. The four exceptions are much larger proteins of *L. plantarum *(LPL1446, LPL3412) and *E. faecalis *(EF0405, EF0406) that have the C-terminal WxL1 domain in common; the larger N-terminal parts of these *L. plantarum *proteins are similar to each other, but have no known other domains, whereas the two *E. faecalis *proteins are also similar to each other and have L-domain-like repeats (see below).

#### CscC family

The CscC family members are much larger than CscA or CscB proteins, and more heterogeneous in size (500–900 residues, with some exceptions). They are multi-domain proteins, all characterized by a C-terminal domain of about 130–140 residues, defined as the "WxL2" domain since it is very similar to the WxL1 domain but differs in overall size, in conserved residues and in the distance between the two WxL motifs (see full alignment of WxL2 domains in additional file 8). Based on these differences, the WxL1 and WxL2 domains can be distinguished as different domain variants, which is also supported by Hidden Markov Models: CscB proteins were recognized by a Hidden Markov model based on the WxL1 domain without false positive hits in CscC proteins, and vice versa.

In addition, other domains could be identified in some CscC proteins with homology to different kinds of binding domains, albeit often with weak homology (see additional file 4). The clearest domain-homologue identified is an N-terminal domain of about 300 residues with structural similarity to concanavalin A-like lectins/glucanases. This superfamily includes a diverse range of carbohydrate-binding domains and glycosyl hydrolase enzymes that share a common structural fold (see Pfam clan CL0004) [36-38]. Lectins and glucanases exhibit the common property of reversibly binding to specific (complex) carbohydrates. This ConA-like domain was found in ten CscC proteins from six different species, and is characterized by several conserved aromatic residues, most of which are tryptophans (see full sequence alignment in additional file 9). Aromatic residues of starch-binding domains have been shown to be involved in the binding of saccharide rings by stacking with indole and phenyl rings [39]. Various (semi)-conserved Asp and Glu residues are potential metal ion ligands, including an ExD motif, as also found in glycosyl hydrolases of this superfamily (see Pfam clan CL0004). The ConA-like domains of CscC proteins show distinct sequence similarity to each other, but much less to other families of the large concanavalin A-like lectin/glucanase superfamily, suggesting that they may represent a new subfamily. The best sequence similarity is with leguminous plant lectins, including the known metal ion binding residues (alignment in additional file 13).

#### CscD family

The CscD family is not characterized by sequence similarity, but rather by the presence of both a signal peptide for secretion, and by an LPxTG-type motif for covalent anchoring to the peptidoglycan matrix. CscD proteins form a very unusual group among the LPxTG-proteins [14], [40], since they are extremely short (90–140 residues) and have only 40–60 residues between the signal peptide (which is removed by signal peptidase I) and the LPxTG-anchoring motif (which is cleaved by sortase). This implies that only a short peptide of that length would become attached to the peptidoglycan. These peptides have very low sequence homology to each other, and multiple sequence alignment is not informative. We propose that they play a role in anchoring the other Csc proteins to the cell surface through as yet unknown interactions.

### Cluster evolution

Family tree analysis of the CscA, CscB and CscC proteins (see additional files 10, 11, 12) suggests first that the clusters have evolved as units without shuffling, as the three trees are basically the same. Secondly, some cluster duplications are of early origin as they precede several speciation events. Other cluster duplications are of more recent origin, as cluster members from the same species are grouped in the same branch, as can be clearly seen in species with many clusters, i.e. *L. plantarum*, *L. sakei, E. faecalis *and *L. lactis*. Also, the gene order in clusters of these more recent duplications has changed little, compared to older duplications (see additional file 3). Finally, multiple copies of *cscB *genes in clusters appear to be the most recent duplications, as they are most similar to members within the same cluster (see additional files 1, 11).

### Co-expression and regulation of cluster genes

Several previous transcriptome investigations aimed at elucidation of *L. plantarum *response under various stress conditions have indicated that the transcription of specific *csc *genes is regulated in response to bile, salt and lactate stress [41,42]. In several cases, the expression of entire *csc *gene clusters was observed to change significantly.

**Figure 3:**
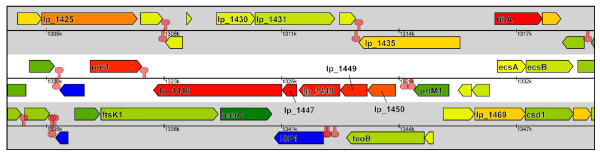
**Example of coordinated gene expression of *csc *gene clusters found in *Lactobacillus plantarum *WCFS1**. Cluster LPL-I (genes lp1446–lp1450) is shown with flanking genes. Genes are color-coded according to relative gene expression (measured by transcriptome analysis) in a comparison of the wild-type strain and a *ccpA *knock-out mutant upon growth on glucose. A sliding color scheme is used from down regulation (dark green) to up regulation (dark red) of genes in mutant *vs *wild type. Blue genes were not measured. Predicted terminators are indicated by loop symbols. Figure were made with the Microbial Genome Viewer [79, 80].

In the present study, seven of the nine *csc *gene clusters of *L. plantarum *appeared to be significantly up-regulated as a consequence of a replacement mutation in the *ccpA *gene (encoding catabolite control protein A, CcpA) when grown on glucose as the main energy and carbon source (Table 2; Figure 3). These data strongly suggest that these gene clusters are part of the catabolite control regulon that is controlled by the central regulator CcpA. To further substantiate this, a MAST-motif search was performed to identify putative CRE sites, for binding of CcpA [43,44], within the *csc *gene clusters and their upstream regions. Putative CRE sites could be identified for six out of the seven up-regulated *csc *clusters, generally upstream of the first gene of the cluster and in three clusters also inside *csc *genes (Figure 1, Table 3). In contrast, no significant CRE-like sites could be identified within or upstream of the residual *csc *gene clusters, supporting a functional role of the identified CRE-site candidate sequences in regulation of these clusters.

**Table 2:** Gene expression data of *L. plantarum*, growth of *ccpA *mutant vs wild-type

				***ccpA *mutant/wild-type**	
				
**cluster**	**gene**	***Csc *family**	**product**	**M**^a^	**P-value**
I	LPL1450	B	extracellular protein	1.00	0.0008
	LPL1449	B	extracellular protein	1.22	0.0003
	LPL1448	A	cell surface protein precursor	1.24	0.0002
	LPL1447	D	cell surface protein precursor	1.36	0.0002
	LPL1446	B	extracellular protein	1.20	0.0006
					
II	LPL2175	B	extracellular protein	1.35	0.0005
	LPL2174	A	cell surface protein precursor	0.79	0.0048
	LPL2173	C	extracellular protein	0.71	0.0032
					
III	LPL2978	B	extracellular protein	0.08	0.7133
	LPL2977	A	cell surface protein precursor	0.90	0.0005
	LPL2976	D	cell surface protein precursor	0.59	0.0077
	LPL2975	C	extracellular protein	0.37	0.0466
					
IV	LPL3067	B	extracellular protein	2.00	0.0001
	LPL3066	A	cell surface protein precursor	1.85	0.0001
	LPL3065	D	cell surface protein precursor	1.49	0.0002
	LPL3064	C	extracellular protein	1.51	0.0001
					
V	LPL3075	C	cell surface protein (putative)	-0.16	0.0780
	LPL3074	D	cell surface protein precursor	0.32	0.0357
	LPL3073	B	extracellular protein	0.01	0.0600
	LPL3072	A	cell surface protein precursor	-0.68	0.0016
					
VI	LPL3117	C	cell surface protein (putative)	1.54	0.0002
	LPL3116	B	extracellular protein	1.76	0.0002
	LPL3115	A	cell surface protein precursor	1.85	0.0000
					
VII	LPL3414	B	extracellular protein	1.21	0.0006
	LPL3413	A	cell surface protein precursor	0.86	0.0079
	LPL3412	B	extracellular protein	0.63	0.0056
					
VIII	LPL3454	D	cell surface protein (putative)	0.67	0.0087
	LPL3453	B	extracellular protein	0.79	0.0013
	LPL3452	B	extracellular protein	1.26	0.0002
	LPL3451	A	cell surface protein precursor	1.13	0.0008
	LPL3450	C	extracellular protein	1.46	0.0006
					
IX	LPL3679	B	extracellular protein	-0.17	0.0159
	LPL3678	A	cell surface protein precursor	0.12	0.1040
	LPL3677	D	cell surface protein precursor	0.49	0.0220
	LPL3676	C	extracellular protein	0.70	0.0018

^a ^M = ^2^log of the expression ratio's, calculated as the average expression level observed in the *ccpA *mutant divided by the average expression ratio observed in the wild-type.

**Table 3:** Putative CRE sites of *L. plantarum csc *clusters

**cluster**	**gene**	**position**	**sequence**	**E-score**
I	LPL1450	starts 66 and 42 bp upstream of gene	**TGATTATCGTTACCA** **TGATCACCGCAGGCA**	n.a.
	LPL1449	inside gene	**TGTAAGCGTCACCA**	3,60E-05
	LPL1446	inside gene	**TGGAACCGCTGGCA**	6,80E-06
				
II	LPL2175	starts 71 bp upstream of gene	**TGAAAGCGGAATCA**	2,60E-05
				
III	LPL2977	inside gene	**TGATAACGGCATCA**	5,00E-06
				
IV	LPL3067	starts 263 bp upstream of gene	**TGTAACCGTTATCC**	8,80E-05
	LPL3066	inside gene	**TGGAACCCTTAACA**	6,30E-05
	LPL3064	inside gene	**TGCAAGCGTATCCA**	1,60E-06
				
V	none			
				
VI	LPL3117	starts 62 and 31 bp upstream of gene	**TGTGAGCGCTATCA** **AGATTACGCTGTCA**	7,80E-06 7,80E-05
				
VII	none			
				
VIII	LPL3454	starts 121 bp upstream of gene	**TGGAATCGCTGTCA**	1,20E-05
				
IX	none			
				
consensus *Bacillus*			**TGAAAGCGTTTTCA**	

n.a. = clear palindromes which are possible CRE sites, but score is lowered due to insertion of 1 extra nucleotide

Taken together these data strongly support the consistent coordinated expression of the *L. plantarum csc *clusters, while a putative role for specific subsets of these clusters in stress survival/adaptation or in carbon source acquisition can be anticipated.

## Discussion

Conserved gene clusters encoding extracellular proteins belonging to four distinct new families have been found in several gram-positive bacteria. Based on the experimental evidence and predictions provided above that the CscA, CscpB, CscC and CscD proteins are functionally coupled, we propose that they form a cell-surface protein complex. Two components are presumably bound to cell-wall components, i.e. the CscA is membrane-anchored and CscD is bound to peptidoglycan. The CscB and CscC proteins have novel WxL domains which could function in binding to CscA/CscD proteins, or to other components of the cell-surface (peptidoglycan, polysaccharides, teichoic acids, etc). The occurrence of these *csc *clusters in a limited number of gram-positive bacteria suggests a niche adaptation. All of the species in Table 1 are free-living bacteria found in the environment. Several of these bacteria are known to be associated with plants and plant fermentations, and many are used for making a variety of fermented products such as sauerkraut, sourdough, olives, silage, soy milk, wine and cheese, or can be found as contaminants of these products. *L. sakei *is more often associated to meat products [32]. It is noteworthy that these gene clusters are neither present in the many sequenced genomes of (mostly pathogenic) streptococci, staphylococci, and clostridia, nor in the *Lactobacillus acidophilus *subgroup of the lactobacilli, which are typical gut bacteria.

Experimental characterization of a Csc family protein has demonstrated its cell-surface location [45]. A *cscB *gene product called Cpf (Co/aggregation-Promoting Factor) of *Lactobacillus coryniformis *DSM20001^T^, a species commonly found in agricultural habitats and food products, was purified and found to mediate coaggregation with and aggregation of other bacterial species. Cpf could be removed from the surface of *Lactobacillus *cells by treatment with high salt (5 M LiCl), and could subsequently be reattached by removal of salt resulting in restoration of the co/aggregation property. This indicates that CscB proteins are non-covalently bound to the bacterial cell surface, supporting our hypothesis.

Transcriptomics experiments show that at least six of the *csc *gene clusters of *L. plantarum *are under catabolite repression, as they are up-regulated in a *ccpA*-knockout strain grown on glucose, and they contain CRE elements for binding of the global regulator CcpA. This regulatory clue suggests a functional link of the Csc proteins with sugar metabolism. Furthermore, some CscC proteins contain ConcanavalinA-like lectin/glucanase domains. ConA-like domains are often found in proteins involved in cell recognition and adhesion, and lectins and glucanases are known to reversibly bind to specific complex carbohydrates. Bacterial and fungal glucanases and xylanases with ConA-like domains can degrade complex polysaccharides like beta-glucans, kappa-carrageenans, xylans and cellulose [36-38,46]. Hence, the presence of ConA-like domains in CscC proteins would support a role of the proposed Csc cell-surface protein complex in binding and/or degradation of complex (plant-derived) oligo- or poly-saccharides. Plant cell-wall polysaccharides are an abundant source of carbon and energy for many free-living micro-organisms, which exploit such polysaccharides from decaying plant material, i.e. in compost, soil, and sewage.

It is striking that the genome of *Lactobacillus plantarum *has the most *csc *gene clusters. *L. plantarum *is frequently found on plants [21,23] and fermented plant material [47], and it is used in plant fermentations [48,49] and silage [22,24]. On plant surfaces, *L. plantarum *should be in close association with other bacteria (or fungi) which are capable of plant polysaccharide degradation and *L. plantarum *could make use of the liberated oligosaccharide units. In addition, or alternatively, *L. plantarum *could have its own extracellular enzyme systems for breakdown of complex polysaccharides, and we hypothesize that the newly described Csc system could be one of such systems.

Extracellular protein complexes for degradation of complex polysaccharides are already known in other groups of bacteria, but they are completely different in protein composition from the putative Csc protein complexes. Some anaerobic bacteria such as *Clostridium *and *Ruminococcus *have an elaborate system called the cellulosome, a large extracellular enzyme complex, to break down plant cell walls. In clostridia, the components of cellulosomes are encoded in large gene clusters [50-52], which are coordinately expressed and regulated by catabolite repression [53]. *Bacteroides thetaiotaomicron*, found in the distal intestine (colon) of the GI-tract, has an outer-membrane-associated multi-protein complex called the starch-utilization system (Sus), consisting of different starch-binding proteins and sugar degradation enzymes encoded in gene clusters [54-57]. Hence, it is not unlikely that during evolution different extracellular protein complexes have arisen in subgroups of bacteria, each specific for a particular environmental niche with its characteristic carbohydrate sources.

## Conclusion

We have presented bioinformatics and experimental evidence that the extracellular CscA, CscB, CscC and CscD proteins are functionally coupled and possibly form a cell-surface protein complex that could play a role in sugar acquisition. Based on the occurrence of these gene clusters in many environmental Gram-positive bacteria, we postulate a role in degradation and utilization of (complex) plant polysaccharides, and possibly other food polysaccharides. Our hypotheses provide a guide for experimental work in any of these bacteria to investigate the location and composition of these protein complexes, their polysaccharide specificity and degradation properties, or the effect of knock-out mutants on the survival of the strain(s) grown on different substrates.

## Methods

### Bioinformatics analysis

Sequence information was obtained from the NCBI bacterial genome database [58] and the ERGO database [59]. The ERGO gene nomenclature was used; conversions to SwissProt nomenclature, where possible, is provided in additional file 5. Genome context was visualised in ERGO and with the Artemis viewer [60]. Terminators were determined with TransTerm [61]. Multiple alignments were created using ClustalW [62] and MUSCLE [63]. Signal peptides were predicted with SignalP [64], and transmembrane helices were detected with TMHMM 2.0 [65]. Conserved sequence patterns and novel domains and motifs were identified with MEME [66] and MAST [67]. Previously described domains were identified by scanning protein sequences with Hidden Markov Models (HMMs) from the PFAM [68], SMART [69] and SUPERFAM [70] databases using the HMMER package. HMMs were compared with HHsearch [71]. Protein family trees were made with LOFT (Rene van der Heijden, personal communication).

Motifs representing catabolite-responsive elements (CRE) were searched by first constructing a MEME profile [66] using 22 established CRE-containing sequences from *B. subtilis *[44]. With this profile, the program MAST [67] was used to detect CRE sites in the *L. plantarum *WCFS1 genome.

Members of the Csc families (see below) were searched for in the NCBI and ERGO databases using BLASTP and Hidden Markov Models (HMMs), starting with the *L. plantarum *Csc protein sequences as seeds, followed by iterative rounds of searches until saturation was reached. Subsequently, we used gene context to search the neighborhood of identified *csc *genes to find additional members of the *csc *gene clusters. This step involved searching in the encoded proteins for signal peptides, LPxTG-type anchoring motifs, and domains containing the WxL motifs (using Hidden Markov Models). In several cases, the correct CDSs were only found after making corrections for missed ORFs, incorrect start codons, frame shifts, etc (see additional files 1, 2).

### Strains, growth conditions, and transcriptome profiling

*L. plantarum *strain LM3 [72] is a close relative of the sequenced strain WCFS1 [31,35] and previous CGH analyses have shown that DNA microarrays based on the genome of strain WCFS1 can be used for transcriptome profiling in this strain: 92% of the probes on the array hybridized with LM3 DNA (D. Molenaar, unpublished data; [35]). Strain LM3 appears to contain all nine *csc *clusters that were identified in the WCFS1 genome, as concluded from array-based genotyping efforts [35] The LM3 strain was used in these studies because a *ccpA*-mutant derivative of this strain is available, LM3-2 (*ccpA::cat*) [72]. Both the parental strain LM3 and its *ccpA *derivative LM3-2 were grown in the 0.25 × MRS medium (prepared without carbon source; [42]) supplemented with 2% glucose. The 1 liter vessel chemostat (Applikon Dependable Instruments, Schiedam, The Netherlands) was operated with 500 ml working volume at 37°C, pH 6.0, 125 rpm, and a flow rate of 120 ml h^-1 ^[73]. The aerobic condition was maintained by sparging the vessel with air at a rate of 29 ml min^-1^. The culture pH was controlled automatically by the addition of 0.5 N HCl or 0.5 N NaOH. The cultures were inoculated with 20 ml of an overnight culture and grown as a batch culture until mid-exponential phase, when continuous feeding of fresh medium was initiated. Samples for RNA extraction were drawn when steady state was reached, that was assumed to require five residence times.

In order to avoid degradation, conversion and *de novo *synthesis of mRNA molecules during sampling of cell culture, we performed a quenching method for collection and centrifugation of cells [74]. Cell pellet was resuspended in TE buffer and transferred in a chilled 2-ml microcentrifuge tube containing 1 g of 0.1-mm-diameter zirconium beads (Biospect Products), 0.25 g macaloid (Kronos Titan GmbH, Leverkusen), 50 μl SDS 10% and 500 μl phenol. The cells were broken by bead-beating [75] at room temperature for 4 times 30 sec, with intermittent cooling on ice for 3 min. After centrifugation for 10 min at 14,500 × g at 4°C, phenol-chloroform extraction was performed until the water phase was clear. RNA was precipitated overnight at 20°C with 1 volume isopropanol, pelleted by centrifugation at 14,500 × g, 20 min, at 4°C, washed once with 70% ethanol and resuspended in appropriate volume of RNase-free MQ-water. Contaminating chromosomal DNA was removed by digestion with RNase-free RQ1 DNase (1 U/μl; Promega) for 15 min at 37°C followed by RNA precipitation with 0.3 M Na-acetate and two volumes of ethanol. The pellet was resuspended in RNase-free MQ-water and determination of sample concentration and quality was performed by an A_260 _and A_280 _reading and by agarose gel electrophoresis. RNA preparations were stored at -80°C until used.

RNA samples were labelled according to previously described methods. The labelled RNA samples were hybridized to previously described, clone-based DNA microarrays that cover more than 80 % of the *L. plantarum *WCFS1 genome, representing 88% of the annotated open reading frames [35]. Hybridizations and washing of the slides, as well as scanning and primary data analyses were performed as previously described.

### Statistical analysis

Microarrays containing fragments of the *L. plantarum *WCFS1 genome as probes were used to measure the expression of genes. The design and production of these arrays as well as the normalization of spot data was described before [76]. Statistical analysis of the data was performed using the "limma" package for R [77,78]. Averaging of spot data to obtain gene-related data was performed as described before [76]. The eBayes function in the limma package was applied to obtain a cross-probe variance estimation and false discovery rate corrected p-values for the whole set of probes. The weighted geometric mean of the false-discovery rate (FDR) corrected p-values was calculated as an indication of significance, although these means do not equal FDR corrected p-values anymore for the complete list of genes.

## Abbreviations

BLASTP Basic Local Alignment Search Tool for Proteins

CRE Catabolite Responsive Element

HMM Hidden Markov Model

MAST Motif Alignment and Search Tool

MEME Multiple Em for Motif Elicitation

PFAM Protein Family database

SMART Simple Modular Architecture Research Tool

TMHMM TransMembrane Hidden Markov Model

## Authors' contributions

RS conceived of the study, participated in its design and coordination, and drafted the manuscript. LM performed the growth studies and microarray experiments, DM performed the statistical analysis, and MK supervised experimental work. JB, BR and RS performed the genome data mining and other bioinformatics sequence analyses. MK and JB contributed to drafting the manuscript and revising it critically for intellectual content.

## Supplementary Material

Additional file 1 Table 4: Summary of *csc *genes and gene clusters. Click here for file

Additional file 2 Table 5: Predicted properties of encoded Csc proteins. Click here for file

Additional file 3 Table 6: Order of genes in cell-surface clusters. Click here for file

Additional file 4 Table 7: Putative other domains in CscB and CscC proteins. Click here for file

Additional file 5 Table 8: Conversion of ERGO codes to SwissProt codes. Click here for file

Additional file 6 Figure 4: Multiple sequence alignment of CscA proteins. Click here for file

Additional file 7 Figure 5: Multiple sequence alignment of CscB proteins. Click here for file

Additional file 8 Figure 6: Multiple sequence alignment of CscC proteins. Click here for file

Additional file 9 Figure 7: Multiple sequence alignment of ConA-like lectins/glucanases domains of CscC proteins. Click here for file

Additional file 10 Figure 8: Family tree of CscA proteins. Click here for file

Additional file 11 Figure 9: Family tree of CscB proteins. Click here for file

Additional file 12 Figure 10: Family tree of CscC proteins. Click here for file

Additional file 13 Figure 11: Multiple sequence alignment of ConA-like lectins/glucanases domains of CscC proteins with known 3D structures of lectins. Click here for file

Additional file 14 Legends to additional Figures. Click here for file
