# Supplementary material for: Lactobacillus plantarum gene clusters encoding putative cell-surface protein complexes for carbohydrate utilization are conserved in specific gram-positive bacteria
Source: BMC Genomics. 2006 May 24;7:126. doi: 10.1186/1471-2164-7-126 (PMC1534035; doi:10.1186/1471-2164-7-126)
Supplement: Additional file 1 — Table 4: Summary of csc genes and gene clusters. [file 1471-2164-7-126-S1.pdf]

Table 4: Summary of *csc* genes and gene clusters.
Gene numbering is according to ERGO. Table 8 contains conversions to SwissProt numbers.

Complete genomes	ERGO code	Cluster-number	*cscC*	*cscD*	*cscA*	*cscB*	comments
L.plantarum WCFS1	LPL		0297				
		I		1447	1448	1446/1449/1450	
		II	2173		2174	2175	
		III	2975	2976	2977	2978	
		IV	3064	3065	3066	3067*	*contains stop codon
		V	3075	3074	3072	3073	
		VI	3117		3115	3116	
		VII			3413	3412/3414	
		VIII	3450	3454	3451	3452/3453	
		IX	3676	3677	3678	3679	
L.monocytogenes EGD-e	LMO	I	0549	0550	0552	0551	
		II	0587		0586	0585	
L.innocua Clip 11262	LIN	I	0552	0553	0555	0554	
		II	0557	0558	0560	0559	
		III	0595		0594	0593	
					0803		
E.faecalis V583	EF	I		0340	0339	0338	
		II		3172	0404	0403/0405/0406	
					0981		
		III	1001	0998	1003	0999/1000/1002	
		IV	1892	-	1894	1893	
						2027/2028/2029	
						2238	
		V	2913A	2913	2289	2287/2288	

		VI	2660	3203	2664	2661/2662/2663	
L.lactis IL1403	LLX	I	1219	1220	1222-1223*	1221	* frameshift
		II		1388	1385-1386*/1389	1387	* frameshift
		III	1616	1621	1614-1615*	1617/1618/1619/1620	* stop codon between 1614 and 1615
B.cereus ZK	BCU	I	0750	0751	0754	0752/753	
B.cereus ATCC10987 #	BCEA	I	0044		0045	0046	on plasmid
B.anthracis A2012	BAT	I	7092-7093*		7091	7090	on plasmid; * frameshift
L.sakei 23K #	LSA	I	0172		0173-0174*/0175	0177	*frameshift
		II	0211-0212*		0213	0215	*frameshift
		III	0613-0614*	0612	0610	0611	*frameshift
		IV	1283	1284	1285-1286c*	1287	* interrupted by IS
		V	1730-1731*		1727-1728*/1729	1725	*frameshift
		VI			1810	1811	
		VII			1816	1815	
		VIII	1820		1818-1819*	1821	*frameshift

**Incomplete genomes**	**ERGO code**	**Cluster-number**	***cscC***	***cscD***	***cscA***	***cscB***	**comments**
L.lactis cremoris SK11	LCR	I	0181		0179	0180	0178 encodes transposase
		II	0187		0188		
		III	0710A	0709A	0709	0710	
		IV		1204A	1206-1207*	1205	small contig; * frameshift
		V		1445		1441/1442/1443/1444	1441 is end of contig
					1867		only gene on contig

P.pentosaceus ATCC25745	PPE	I	0545	0546	0547	0548	
		II			0557	0558	
E.faecium DO	EFA	I	3594	3606	3601	4241	
		II	3967		3978-3983**	3975	** stopcodon between 3978 and 3983
		III	3340		4468	3344-3344A*	* frameshift
L.brevis ATCC367	LBR		0331				small contig
		I	0625			0624	small contig
			0959				
			1027-1028*				small contig, * frameshift
			1269				
L.mesenteroides ATCC8293	LME	I	0911	0910A	0909	0910	909 end of contig, 910A orf missed
L.casei ATCC334	LCA				0104		small contig
		I			0270-0271*	0269	small contig, * N-term missing
		II	0293*	0294	0297	0295/0296	contig is 293-298, * N-term missing
		III	0329	0327A		0327*/0328	small contig, * N-term missing
		IV	0972*	0971	0970		small contig 970-972, * C-term missing
B.thuringiensis ATCC35646	BTH	I	3761	3760	3757	3758/3759	
O.oeni PSU-1	OOE	I			1273-1274*	1275	*frameshift

NCBI numbering
