# Supplementary material for: Lactobacillus plantarum gene clusters encoding putative cell-surface protein complexes for carbohydrate utilization are conserved in specific gram-positive bacteria
Source: BMC Genomics. 2006 May 24;7:126. doi: 10.1186/1471-2164-7-126 (PMC1534035; doi:10.1186/1471-2164-7-126)
Supplement: Additional file 2 — Table 5: Predicted properties of encoded Csc proteins. [file 1471-2164-7-126-S2.pdf]

Table 5: Predicted properties of encoded Csc proteins
[a] New residue length after start correction; old length in parenthesis.
n.a. not applicable

CscA

	ERGO gene code		AA [a]	signal peptide	gene cluster	pI	Comments
L.plantarum WCFS1	LPL	1448	348	+	1446-1450	10.63	
	LPL	2174	349	+	2173-2175	11.31	
	LPL	2977	347	+	2975-2978	10.56	
	LPL	3066	354	+	3064-3067	10.85	
	LPL	3072	347	+	3069-3075	10.72	
	LPL	3115	353	+	3115-3117	10.74	
	LPL	3413	344	+	3412-3414	10.73	
	LPL	3451	337	+	3450-3454	10.53	
	LPL	3678	353	+	3676-3679	11.10	
L.monocytogenes EGD-e	LMO	0552	343	+	0552-0555	8.72	
	LMO	0586	341	+	0585-0587	9.59	
L.innocua Clip 11262	LIN	0555	343	+	0551-0560	6.2	
	LIN	0560	343	+	0551-0560	8.79	
	LIN	0594	341	+	0593-0595	9.74	
	LIN	0803	330	+			
E.faecalis V583	EF	0339	377	+	0338-0340	8.76	
	EF	0404	349	+	403-406,3172	10.26	
	EF	0981	354	+		9.58	

	EF	1003	364	+	0998-1003	9.62	
	EF	1894	344(314)	+	1892-1894	7.23	new start at nucleotide 665685 (ATG)
	EF	2289	341	+	2913,2287-2289	4.94	
	EF	2664	360(358)	+	2660-2666,3203	7.56	new start at nucleotide 3056841 (ATG)
B.anthracis A2012	BAT	7091	360(362)	?	7090-7093	9.27	new start at nucleotide 78625 (ATG)
L.lactis IL 1403	LLX	1222	189(215)	+	1219-1223	10.27	new start at nucleotide 1220573 (ATG),frameshift with 1223
	LLX	1223	187	n.a.	1219-1223	9.71	frameshift with 1222
	LLX	1385	108	n.a.	1385-1389	9.89	frameshift with 1386
	LLX	1386	289	+	1385-1389	5.48	frameshift with 1385
	LLX	1389	334	+	1385-1389	9.32	
	LLX	1614	259	n.a.	1614-1621	9.51	stopcodon between 1614 and 1615
	LLX	1615	124	+	1614-1621	4.77	stopcodon between 1614 and 1615
B. cereus ZK	BCU	0754	341	+	0750-0754	7.61	
B. cereus ATCC10987	BCEA	0045	361	?	0044-0046		plasmid; NCBI gene code
L.lactis cremoris SK11	LCR	0179	376	+	0179-0181	9.79	
	LCR	0188	333	+	0187-0188	9.89	
	LCR	0709	376	+	0709-0710	9.30	
	LCR	1206	319	+	1204A-1207	9.27	frameshift with 1207
	LCR	1207	68	n.a	1204A-1207	9.78	frameshift with 1206
	LCR	1867	336	+		9.88	

P.pentosaceus ATCC25745	PPE	0547	352	+	545-548	10.86	
	PPE	0557	323	+	557-558	10.72	
E.faecium DO	EFA	3601	344	+	3601,3606,3594,4241	4.93	
	EFA	3978	181	+	3972-3983	7.60	stopcodon between 3978 and 3983
	EFA	3983	151	n.a	3967-3983	9.87	stopcodon between 3978 and 3983
	EFA	4468	358	+	880-3344A	4.87	
L.mesenteroides ATCC8293	LME	0909	276	+	0909-0911	9.68	C-term missing, end contig
L.casei ATCC334	LCA	0104	344	+	-	10.33	
	LCA	0270	360	+	0269-0271	10.99	
	LCA	0271	287	-	0269-0271	10.36	N-term missing, no RBS
	LCA	0297	359	+	0293-0298	9.90	
	LCA	0970	336	+	0970-0972	10.17	
B.thuringiensis ATCC35646	BTH	3757	247	+	3757-3761	5.54	C-terminally truncated; end of contig
O.oeni PSU-1	OOE	1273	164	n.a	1273-1275	11.11	frameshift with 1274
	OOE	1274	166	+	1273-1275	11.25	frameshift with 1273
L.sakei 23K	LSA	0173	259	+	0172-0177		frameshift with 0174
		0174	90	n.a.	0172-0177		frameshift with 0173
		0175	342	+	0172-0177		
		0213	345	+	0211-0215		
		0610	351	+	0610-0614		
		1285	193	n.a.	1283-1287		separated from 1286c by IS

		1286c	158	+	1283-1287		separated from 1285 by IS
		1727	82	n.a.	1725-1731		frameshift with 1728
		1728	272(262)	+	1725-1731		new start at 1709904(ATG), frameshift with 1727
		1729	345(327)	+	1725-1731		new start at 1710946(GTG)
		1810	350(335)	+	1810-1811		new start at 1802205(ATG)
		1816	337	+	1815-1816		
		1818	28	n.a.	1818-1821		frameshift with 1819
		1819	293	+	1818-1821		frameshift with 1818

CscB

	ERGO gene code		AA [a]	signal peptide	gene cluster	pI	Comments
L.plantarum WCFS1	LPL	1446	1092	+	1446-1450	4.85	
	LPL	1449	226	+	1446-1450	7.51	
	LPL	1450	234	+	1446-1450	4.14	
	LPL	2175	243	+	2173-2175	4.65	
	LPL	2978	260	+	2975-2978	4.38	
	LPL	3067	234	+	3064-3067	4.37	contains stopcodon, new stop at 2729791
	LPL	3073	202	+	3069-3075	4.65	
	LPL	3116	190	+	3115-3117	4.84	
	LPL	3412	1136	+	3412-3414	4.91	
	LPL	3414	269	+	3412-3414	4.66	
	LPL	3452	230	+	3450-3454	4.91	
	LPL	3453	190	+	3450-3454	4.37	
	LPL	3679	245	+	3676-3679	4.24	
L.monocytogenes EGD-e	LMO	0551	280	+	0549-0552	4.14	

	LMO	0585	237	+	0585-0587	4.33	
L.innocua Clip 11262	LIN	0554	280	+	0552-0555	4.25	
	LIN	0559	280	+	0557-0560	4.55	
	LIN	0593	237	+	0593-0595	4.43	
E.faecalis V583	EF	0338	229	+	0338-0340	4.31	
	EF	0403	222	+	0403-0406,3172	4.68	
	EF	0405	711(647)	+	0403-0406,3172	4.42	new start at nucleotide 2174870
	EF	0406	1004	+	0403-0406,3172	4.2	different and longer N-term
	EF	0999	243	+	0998-1003	4.54	
	EF	1000	259	+	0998-1003	5.13	
	EF	1002	246	+	0998-1003	4.28	
	EF	1893	245	+	1892-1894	7.67	
	EF	2027	232	+	2027-2029	4.54	
	EF	2028	236	+	2027-2029	4.41	
	EF	2029	238	+	2027-2029	4.41	
	EF	2238	239	+	2238	4.71	
	EF	2287	260	+	2913,2287-2289	4.39	
	EF	2288	258	+	2913,2287-2289	4.55	
	EF	2661	244	+	2660-2666,3203	4.65	
	EF	2662	266	+	2660-2666,3203	4.57	
	EF	2663	252	+	2660-2666,3203	4.61	
B.anthracis A2012	BAT	7090	200(202)	+	7090-7093	9.68	new start at nucleotide 77949 (ATG)
L.lactis IL 1403	LLX	1221	176	+	1219-1223	5.66	

	LLX	1387	270	+	1385-1389	4.81	
	LLX	1617	251	+	1614-1621	4.22	
	LLX	1618	252	+	1614-1621	8.76	
	LLX	1619	263(143)	+	1614-1621	4.97	new start at nucleotide 1626944 (ATG) with SP in other frame
	LLX	1620	281	+	1614-1621	4.09	
B.cereus ZK	BCU	0752	261	+	0750-0754	4.58	
	BCU	0753	251	+	0750-0754	4.76	
B.cereus ATCC10987	BCEA	0046	201		0044-0046		plasmid; NCBI gene code
L.lactis cremoris SK11	LCR	0180	238	+	0179-0181	6.52	
	LCR	0710	235(157)	+	0709-0710	4.36	new start at nucleotide 29343 (TAT, ATG in other frame)
	LCR	1205	270	+	1204A-1207	4.65	
	LCR	1441	240	+	1441-1445	4.39	
	LCR	1442	252	+	1441-1445	5.97	
	LCR	1443	263	+	1441-1445	4.19	
	LCR	1444	281	+	1441-1445	4.1	
P.pentosaceus ATCC25745	PPE	0548	217	+	0545-0548	5.6	
	PPE	0558	162	+	0557-0558	4.36	
E.faecium. DO	EFA	3344	191	n.a	0880-3344A	9.52	frameshift with 3344A
	EFA	3344A	80	+	0880-3344A		new orf: start at nucleotide 11891(ATG), stop at nucleotide 11649, frameshift with 3344
	EFA	3975	252	+	3967-3983	4.07	
	EFA	4241	226	+	3601,3606,3594,4241	4.15	

L.brevis ATCC367	LBR	0624	199	+	0623-0625	4.25	
L.mesenteroides ATCC8293	LME	0910	259	+	0909-0911	5.27	
L.casei ATCC334	LCA	0269	299	+	0269-0271	4.64	
	LCA	0295	257	+	0293-0298	4.94	
	LCA	0296	247	+	0293-0298	6.28	
	LCA	0327	220	-	0327-0329	4.45	end of contig; N-terminally truncated ?
	LCA	0328	234	+	0327-0329	9.49	
B.thuringiensis ATCC35646	BTH	3758	249	+	3757-3761	5.00	
		3759	242	+	3757-3761	6.56	
O.oeni PSU-1	OOE	1275	191	+	1273-1275	3.39	
L.sakei 23K	LSA	0177	294	+	0172-0177		
		0215	327	+	0211-0215		
		0611	233	+	0610-0614		
		1287	203	+	1283-1287		
		1725	325	+	1725-1731		
		1811	240	+	1810-1811		
		1815	171(165)	+	1815-1816		new start at 1806685(TTG)
		1821	200	+	1818-1821		

CscC

	ERGO gene code		AA [a]	signal	gene cluster	pI	Comments

				peptide			
L.plantarum WCFS1	LPL	0297	685	+	-	4.89	
	LPL	2173	482	+	2173-2175	5.19	
	LPL	2975	502	+	2975-2978	8.65	
	LPL	3064	495	+	3064-3067	9.70	
	LPL	3075	915	+	3069-3075	8.23	
	LPL	3117	750	+	3115-3117	4.23	
	LPL	3450	716	+	3450-3454	4.45	
	LPL	3676	512	+	`	10.04	
L.monocytogenes EGD-e	LMO	0549	673	+	0549-0552	4.61	
	LMO	0587	821	+	0585-0587	4.33	
L.innocua Clip 11262	LIN	0552	701	+	0552-0555	4.42	
	LIN	0557	664	+	0557-0560	5.30	
	LIN	0595	821	+	0593-0595	4.36	
E.faecalis V583	EF	1001	699	+	0998-1003	4.50	
	EF	1892	761(763)	+	1892-1894	5.42	new start at nucleotide 668843 (TTG)
	EF	2913A	723	+	2913,2287-2289		new orf, start at nucleotide 2599386(ATG), stop at nucleotide 2597215
	EF	2660	1619(1554)	+	2660-2666,3203	4.91	new start at nucleotide 3064471 (ATG)
B. anthracis A2012	BAT	7092	580(582)	+	7090-7093	8.84	new start at nucleotide 79741(ATG), frameshift with 7093
	BAT	7093	95	n.a.	7090-7093	9.93	frameshift with 7092
L.lactis IL 1403	LLX	1219	749	-	1219-1223	4.78	N-terminus interrupted by transposase

	LLX	1616	1649	+	1614-1621	4.73	
B.cereus ZK	BCU	0750	898	?	0750-0754	4.81	
B.cereus ATCC10987	BCEA	0044	646	+	0044-0046		plasmid; NCBI gene code
L.lactis cremoris SK11	LCR	0181	918	+	0179-0181	4.56	
	LCR	0187	700(672)	n.a.	0187-0188	4.41	new start at nucleotide 19868 (TTG), frameshift with 187A
	LCR	0187A	13	+			new orf, start at nucleotide 19647(TTG), stop at nucleotide 19686, frameshift with 0187
	LCR	0710A	915	+	0709-0710		new orf, start at nucleotide 30157(ATG), stop at nucleotide 32904
P.pentosaceus ATCC25745	PPE	0545	681	+	0545-0548	7.18	
E.faecium DO	EFA	3340	675	?	0880-3344A	4.54	
	EFA	3594	826	+	3601,3606,3594,4241	5.16	
	EFA	3967	1055(1036)	+	3967-3983	4.28	new start at nucleotide 23773 (ATG)
L.brevis ATCC367	LBR	0331	729	-		5.01	end of contig; N-terminally truncated
	LBR	0625	715	+	0623-0625	4.50	
	LBR	0959	600	+		5.01	
	LBR	1027	211	n.a.	1027-1028	4.10	frameshift with 1028
	LBR	1028	503	+	1027-1028	4.47	frameshift with 1027
	LBR	1269	707	+		6.27	
L. mesenteroides ATCC8293	LME	0911	510	+	0909-0911	4.97	

L.casei ATCC334	LCA	0293	680	-	0293-0298	6.46	N-term missing; end of contig
	LCA	0329	673	+	0327-0329	7.02	
	LCA	0972	214	+	0970-0972	9.55	C-term missing
B.thuringiensis ATCC35646	BTH	3761	861(683)	+	3758-3761	8.66	new start at nucleotide 5415 (TTG)
L.sakei 23K	LSA	0172	1311(1303)	+	0172-0177		new start at 163816(GTG)
		0211	109(154)	+	0211-0215		new start at 204620(TTG), new stop at 204946, frameshift with 0212
		0212	1044	n.a.	0211-0215		frameshift with 0211
		0613	542	n.a.	0610-0614		frameshift with 0614
		0614	418	+	0610-0614		frameshift with 0613
		1283	611	+	1283-1287		
		1730	625	n.a.	1725-1731		frameshift with 1731
		1731	1768	+	1725-1731		frameshift with 1730
		1820	704	+	1818-1821		

CscD

	ERGO gene code		AA [a]	signal peptide	gene cluster	pI	Comments
L.plantarum WCFS1	LPL	1447	123	+	1446-1450	10.01	
	LPL	2976	125	+	2975-2978	11.23	
	LPL	3065	141	+	3064-3067	11.58	
	LPL	3074	123	+	3069-3075	8.54	
	LPL	3454	92	-	3450-3454	9.34	possible frameshifts at N-terminus
	LPL	3677	113	+	3676-3679	10.18	
L.monocytogenes EGD-e	LMO	0550	98	+	0549-0552	10.31	

L.innocua Clip 11262	LIN	0553	97	+	0552-0555	10.49	
		0558	96	+	0557-0560	10.52	
E.faecalis V583	EF	0340	133(122)	+	0338-0340	10.2	new start at nucleotide 2841213
	EF	0998	112	+	0998-1003	4.84	
	EF	2913	122	+	2913,2287-2289	10.2	
	EF	3172	109	+	0403-0406,3172	11.54	
	EF	3203	129	+	2660-2666,3203	7.18	
L.lactis IL 1403	LLX	1220	111(105)	+	1219-1223	8.5	N-term has frameshift in SP,start at nucleotide 1219654 (ATG)
	LLX	1388	114	+	1385-1389	10.3	
	LLX	1621	101(31)	+	1614-1621	9.93	N-term with SP in other frame, start at nucleotide 1628183 (TTG)
B.cereus ZK	BCU	0751	115	+	0750-0754	8.83	
L.lactis cremoris SK11	LCR	0709A	106	+	0709-0710		missed orf, start at nucleotide 28972(TTG) stop at nucleotide 29292
	LCR	1204A	111	+	1204A-1207		missed orf, start at nucleotide 639(ATG) stop at nucleotide 974
	LCR	1445	99	+	1441-1445	9.84	
P.pentosaceus ATCC25745	PPE	0546	112	+	0545-0548	10.66	
E.faecium DO	EFA	3606	111	+	3601,3606,3594,4241	6.80	
L. mesenteroides ATCC8293	LME	0910A	124	+	0909-0911		missed orf, start at nucleotide 2029(ATG) stop at nucleotide 1640

L.casei ATCC334	LCA	0294	119	+	0293-0298	4.44	
	LCA	0327A	120	+	0327-0329		missed orf, start at nucleotide 986(ATG) stop at nucleotide 1345
	LCA	0971	125	+	0970-0972	7.79	
B.thuringiensis ATCC35646	BTH	3760	105	+	3757-3761	10.68	
L.sakei 23K	LSA	0612	118	+	0610-0614		
		1284	131	+	1283-1287		
