# Supplementary material for: Lactobacillus plantarum gene clusters encoding putative cell-surface protein complexes for carbohydrate utilization are conserved in specific gram-positive bacteria
Source: BMC Genomics. 2006 May 24;7:126. doi: 10.1186/1471-2164-7-126 (PMC1534035; doi:10.1186/1471-2164-7-126)
Supplement: Additional file 3 — Table 6: Order of genes in cell-surface clusters. [file 1471-2164-7-126-S3.pdf]

**Table 6. Order of genes in cell-surface clusters**

| Complete genomes | cluster | *csc* gene order | | | | |
|---|---|---|---|---|---|---|
| L.plantarum WCFS1 | I | B(2) | A | D | B | |
| | II | B | A | C | | |
| | III | B | A | D | C | |
| | IV | B | A | D | C | |
| | V | C | D | B | A | |
| | VI | C | B | A | | |
| | VII | B | A | B | | |
| | VIII | D | B(2) | A | C | |
| | IX | B | A | D | C | |
| L.monocytogenes EGD-e | I | C | D | B | A | |
| | II | B | A | C | | |
| L.innocua Clip 11262 | I | C | D | B | A | |
| | II | C | D | B | A | |
| | III | B | A | C | | |
| E.faecalis V583 | I | B | A | D | | |
| | II | B | A | D | B(2) | |
| | III | D | B(2) | C | B | A |
| | IV | C | B | A | | |
| | V | C | D | B(2) | A | |
| | VI | C | D | B(3) | A | |
| B. anthracis A2012 | I | B | A | C | | |
| L.lactis IL1403 | I | C | D | B | A | |
| | II | A | D | B | A | |
| | III | D | B(4) | C | A | |
| B.cereus ZK | I | C | D | B(2) | A | |
| B.cereus ATCC10987 | I | B | A | C | | |
| L.sakei 23K | I | C | A(2) | B | | |
| | II | C | A | B | | |

| | | | | | |
|---|---|---|---|---|---|
| | III | C | D | B | A |
| | IV | B | A | D | C |
| | V | C | A(2) | B | |
| | VI | B | A | | |
| | VII | B | A | | |
| | VIII | B | C | A | |

| **Incomplete genomes** | **cluster** | ***csc* gene order** | | | |
|---|---|---|---|---|---|
| L.lactis cremoris SK11 | I | A | B | C | |
| | II | C | A | | |
| | III | D | A | B | C |
| | IV | D | B | A | |
| | V | D | B(4) | | |
| P.pentosaceus ATCC25745 | I | B | A | D | C |
| | II | B | A | | |
| E.faecium DO | I | B | A | D | C |
| | II | C | A | B | |
| | III | B | C | A | |
| L.brevis ATCC367 | I | B | C | | |
| L.mesenteroides ATCC8293 | I | C | D | B | A |
| L.casei ATCC334 | I | | A(2) | B | |
| | II | C | D | B(2) | A |
| | III | B | D | B | C |
| | IV | A | D | C | |
| B.thuringiensis ATCC35646 | I | C | D | B(2) | A |
| O.oeni PSU-1 | I | B | A | | |
