# Supplementary material for: Lactobacillus plantarum gene clusters encoding putative cell-surface protein complexes for carbohydrate utilization are conserved in specific gram-positive bacteria
Source: BMC Genomics. 2006 May 24;7:126. doi: 10.1186/1471-2164-7-126 (PMC1534035; doi:10.1186/1471-2164-7-126)
Supplement: Additional file 4 — Table 7: Putative other domains in CscB and CscC proteins. [file 1471-2164-7-126-S4.pdf]

Table 7: Putative other domains in CscB and CscC proteins, in addition to WxL1 (in CscB) and WxL2 (in CscC).

protein	WxL type	other domain code	domain type/function[a]	HMM score[b]	start	stop
EF1001	WxL2	SUPERFAM~49899~0021920	Concanavalin A-like lectins/glucanases [c]	2.8e-31	120	370
EFA3340	WxL2	SUPERFAM~49899~0021920	Concanavalin A-like lectins/glucanases	6.4e-30	91	342
LBR0331	WxL2	SUPERFAM~49309~0021724	Concanavalin A-like lectins/glucanases	7.4e-04	43	336
		SUPERFAM~49309~0021724	Transglutaminase, two C-terminal domain	6.7e-06	482	576
LBR0625	WxL2	SUPERFAM~49899~0024562	Concanavalin A-like lectins/glucanases	2.0e-15	57	340
LBR1027-1028	WxL2	SUPERFAM~0024184~49899	Concanavalin A-like lectins/glucanases	1.7e-14	42	327
LBR1269	WxL2	SUPERFAM~49899~0018963	Concanavalin A-like lectins/glucanases	2.3e-04	38	322
		SUPERFAM~49384~0018753	Carbohydrate-binding domain	1.7e-04	435	526
LCA0329	WxL2	SUPERFAM~49899~0024184	Concanavalin A-like lectins/glucanases	5.3e-08	44	325
LPL0297	WxL2	SUPERFAM~49309~0021724	Concanavalin A-like lectins/glucanases	4.6e-06	53	288
LPL3450	WxL2	SUPERFAM~49899~0024184	Concanavalin A-like lectins/glucanases	2.6e-19	47	335
LSA1820	WxL2	SUPERFAM~49899~0024184	Concanavalin A-like lectins/glucanases	2.2e-19	32	284
EF0406	WxL1	SUPERFAM~52058~0021078	L domain-like[d]	7.1e-40	92	401
EF0405	WxL1	SUPERFAM~52058~0022161	L domain-like	8.7e-19	76	254
LPL3075	WxL2	SUPERFAM~52058~0022972	L domain-like	1.0e-19	155	528
LPL3117	WxL2	SUPERFAM~52058~0023073	L domain-like	7.5e-19	105	490
LPL2975	WxL2	SUPERFAM~81296~0017693	E set domains = "Early" Ig-like fold families	7.2e-04	178	262
LPL3064	WxL2	SUPERFAM~56601~0016598	beta-lactamase/transpeptidase-like	3.2e-03	1	124
LPL3676	WxL2	SUPERFAM~54358~0021100	Immunoglobulin-binding domains	2.7e-03	249	313
LMO0587	WxL2	SUPERFAM~51126~0019984	Pectin lyase-like[c]	5.4e-05	1	279
		SUPERFAM~52047~0024268	RNI-like[d]	8.4e-08	134	234
		SUPERFAM~49401~0016614	Bacterial adhesins	1.0e-13	233	374
		SUPERFAM~53850~0023866	Periplasmic binding protein-like II	2.6e-04	476	538
LIN0595	WxL2	SUPERFAM~51126~0019984	Pectin lyase-like	1.2e-05	1	279

		SUPERFAM~49401~0016614	Bacterial adhesins	5.1e-16	233	374
LCA0293	WxL2	SUPERFAM~51126~0021375	Pectin lyase-like	2.0e-04	131	261
LMO0549	WxL2	SUPERFAM~52047~0024268	RNI-like	4.3e-17	128	276
LIN0552	WxL2	SUPERFAM~52058~0019210	L domain-like	2.2e-14	142	296
		PFAM~Big_3	Bacterial Ig-like domain (group 3)	7.9e-03	479	548
LSA0211-0212	WxL2	PFAM~DUF285	Mycoplasma protein of unknown function, DUF2	5.4e-06	324	480
		SUPERFAM~52058~0022972	L domain-like	1.9e-12	282	565
LSA1730-1731	WxL2	SUPERFAM~49401~0016614	Bacterial adhesins	5.7e-06	91	147
		SUPERFAM~49373~0017678	Invasin/intimin cell-adhesion fragments	2.4e-05	1609	1685
		PFAM~Fil_haemagg	Haemagluttinin repeat	2.8e-05	1255	1319
LPL2173	WxL2	SUPERFAM~48726~0018866	Immunoglobulin	5.2e-09	49	152
		SUPERFAM~49373~0018812	Invasin/intimin cell-adhesion fragments	6.6e-12	145	246
LBR0959	WxL2	SUPERFAM~49373~0018812	Invasin/intimin cell-adhesion fragments	2.2e-03	77	169
LLX1219	WxL2	PFAM~Big_3	Bacterial Ig-like domain (group 3)	3.2e-23	470	536
BAT7092	WxL2	SUPERFAM~49265~0021408	Fibronectin type III	3.5e-04	362	418
LLX1616	WxL2	SUPERFAM~51110~0017993	alpha-D-mannose-specific plant lectins	6.4e-03	984	1102

.

[a] Low-scoring irrelevant domains removed (e.g. Chlamydia polymorphic membrane protein)

[b] Minimal e-score: 0.01

[c] Concanavalin A-like lectins/glucanases and pectin lyase are similar domains

[d] L domain-like & RNI-like are similar domains
