# Supplementary material for: Lactobacillus plantarum gene clusters encoding putative cell-surface protein complexes for carbohydrate utilization are conserved in specific gram-positive bacteria
Source: BMC Genomics. 2006 May 24;7:126. doi: 10.1186/1471-2164-7-126 (PMC1534035; doi:10.1186/1471-2164-7-126)
Supplement: Additional file 5 — Table 8: Conversion of ERGO codes to SwissProt codes. [file 1471-2164-7-126-S5.pdf]

Table 8. Conversion ERGO codes to SwissProt codes

L.plantarum WCFS1		L.monocytogenes EGDe		L.innocua Clip 11262		E.faecalis V583		B.anthracis A2012		L.lactis IL1403		L. sakei 23K		B. cereus 10987	
**LPL**		**LMO**		**LIN**		**EF**		**BAT**		**LLX**		**LSA**		**BCEA**	
0297	Q88ZL6	0549	Q8Y9H9	0552	Q92EA7	0338	Q82ZS7	7090	Q8KYN7	1219	Q9CGA2	0172	Q38ZA8	0044	Q74P50
		0550	Q8Y9H8	0553	Q92EA6	0339	Q82ZS8	7091	Q9X336	1220	Q9CGA1	0173	Q38ZA7	0045	Q74P49
1446	Q88X10	0551	Q8Y9H7	0554	Q92EA5	0340	Q82ZS9	7092	Q9X337	1221	Q9CGA0	0174	Q38ZA6	0046	Q74P48
1447	Q88X09	0552	Q8Y9H6	0555	Q92EA4			7093	Q8KYN6	1222	Q9CG99	0175	Q38ZA5		
1448	Q88X08					3172	Q832H6			1223	Q9CG98	0177	Q38ZA3		
1449	Q88X07	0585	Q8Y9E5	0557	Q92EA2	0403	Q832H4								
1450	Q88X06	0586	Q8Y9E4	0558	Q92EA1	0404	Q832H5			1385	Q9CFW1	0211	Q38Z69	**B. cereus ZK**	
		0587	Q8Y9E3	0559	Q92EA0	0405	Q832H8			1386	Q9CFW0	0212	Q38Z68	**BCU**	
2173	Q88VA1			0560	Q92E99	0406	Q832I0			1387	Q9CFV9	0213	Q38Z67	0750	Q63FE3
2174	Q88VA0									1388	Q9CFV8	0215	Q38Z65	0751	Q63FE2
2175	Q88V99			0593	Q92E67	0981	Q836D8			1389	Q9CFV7			0752	Q63FE1
				0594	Q92E66							0610	Q38Y15	0753	Q63FE0
2975	Q88TI1			0595	Q92E65	0998	Q837T1			1614	Q9CFA4	0611	Q38Y14	0754	Q63FD9
2976	Q88TI0					0999	Q837T0			1615	Q9CFA3	0612	Q38Y13		
2977	Q88TH9			0803	Q92DK9	1000	Q837S9			1616	Q9CFA2	0613	Q38Y12		
2978	Q88TH8					1001	Q837S8			1617	Q9CFA1	0614	Q38Y11		
						1002	Q837S7			1618	Q9CFA0				
3064	Q88TB4					1003	Q837S6			1619	Q9CF99	1283	Q38W48		
3065	Q88TB3									1620	Q9CF98	1284	Q38W47		
3066	Q88TB2/1					1892	Q837W4			1621	Q9CF97	1285	Q38W46		
3067	Q88TB0					1893	Q837W5					1286	Q38W43		
						1894	Q837W6					1287	Q38W42		
3072	Q88TA6														

3073	Q88TA5
3074	Q88TA4
3075	Q88TA3
3115	Q88T69
3116	Q88T68
3117	Q88T67
3412	Q88SK2
3413	Q88SK1
3414	Q88SK0
3450	Q88SH3
3451	Q88SH2
3452	Q88SH1
3453	Q88SH0
3454	Q88SG9
3676	Q88RY0
3677	Q88RX9
3678	Q88RX8
3679	Q88RY7

2027	Q82ZB1
2028	Q82ZB2
2029	Q82ZB3
2238	Q836A2
2287	Q830T7
2288	Q830T8
2289	Q830T9
2913	Q830T6
2660	Q82Z82
2661	Q82Z84
2662	Q82Z85
2663	Q82Z86
2664	Q82Z87
3203	Q82Z83

1725	Q38UV1
1727	Q38UU9
1728	Q38UU8
1729	Q38UU7
1730	Q38UU6
1731	Q38UU5
1810	Q38UL7
1811	Q38UL6
1815	Q38UL2
1816	Q38UL1
1818	Q38UK9
1819	Q38UK8
1820	Q38UK7
1821	Q38UK6
