# Supplementary material for: Lactobacillus plantarum gene clusters encoding putative cell-surface protein complexes for carbohydrate utilization are conserved in specific gram-positive bacteria
Source: BMC Genomics. 2006 May 24;7:126. doi: 10.1186/1471-2164-7-126 (PMC1534035; doi:10.1186/1471-2164-7-126)
Supplement: Additional file 6 — Figure 4: Multiple sequence alignment of CscA proteins. [file 1471-2164-7-126-S6.pdf]

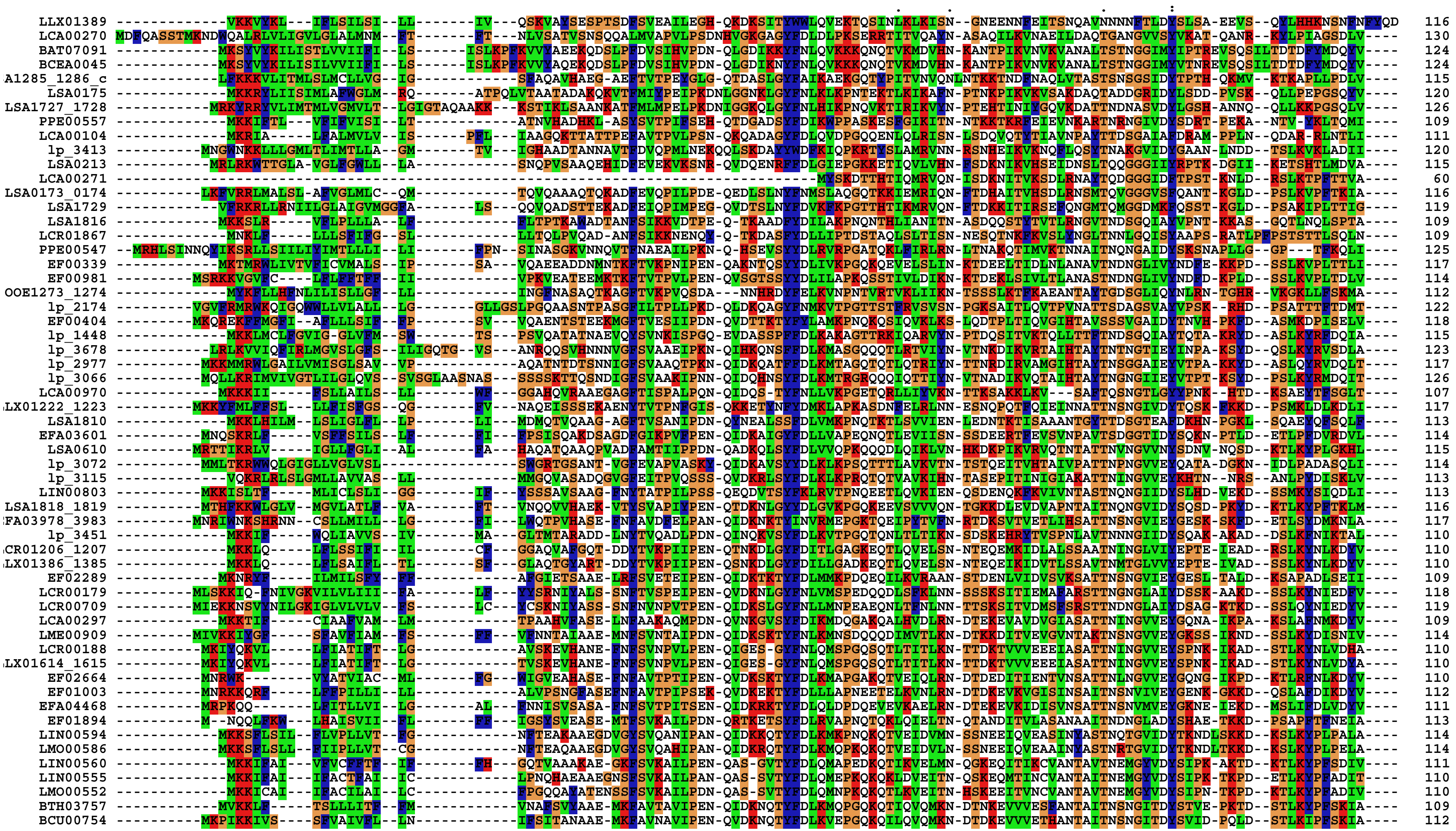
LLX01389 116
LCA00270 130
BAT07091 124
BCEA0045 124
A1285_1286_c 115
LSA0175 120
LSA1727_1728 126
PPE00557 109
LCA00104 111
lp_3413 120
LSA0213 115
LCA00271 60
LSA0173_0174 116
LSA1729 119
LSA1816 109
LCR01867 109
PPE00547 125
EF00339 117
EF00981 114
OOE1273_1274 112
lp_2174 122
EF00404 118
lp_1448 115
lp_3678 123
lp_2977 117
lp_3066 126
LCA00970 107
LX01222_1223 117
LSA1810 113
EFA03601 114
LSA0610 115
lp_3072 114
lp_3115 113
LIN00803 113
LSA1818_1819 116
FA03978_3983 117
lp_3451 110
CR01206_1207 110
LX01386_1385 110
EF02289 109
LCR00179 118
LCR00709 119
LCA00297 109
LME00909 114
LCR00188 110
LX01614_1615 110
EF02664 110
EF01003 112
EFA04468 111
EF01894 113
LIN00594 114
LMO00586 114
LIN00560 111
LIN00555 110
LMO00552 110
BTH03757 109
BCU00754 112

```
                         :                                                     .                           .                          .  *
LLX01389      IYFDETQEAGKLKITLAPNEVREITIN-LKIPKEGIKGQLIGGINVTKVPKESDRQ-------EGILNVYSNVVALVME-DKDYSESHKQNLEFALEKSNEKEQ------VIKIKNSNSFLLSNMNIKAVIKDQKG-EVVSEFSNPKTAV   250
LCA00270      -----KID---RQFELKPGETRQLPIV-LKPGKGQPRGTYLSAIRLSALAPSTTDT--------SVQNRIAYTLGLVLHNGHVAG--SVSNLRMGQTSLVQKRS--GFQLRSQVQNPKRIFLKDVSAKINLTSATSRFFSLTVTRELPRI   259
BAT07091      -----QAP---DAVTLEENQTKTIEVH-LTAPNQ--GGVFLGGLLFELAHDTQGQKLEKDGLNFSVHNRIEYAVAIQLNSEETENSVNKAPLKLNGTTVESYPT--QPQVHALFQNTNANIVRDVAVYYEVLSQD--NVLFHGNIPAFNV   259
BCEA0045      -----QAP---DAVNLEGNQTKTIEVH-LTTPNQ--DGVFLGGLLFELAHDTQGQKLEKDGLNFSVHNSIEYAVAIQLNSEETENSVNKTPLKLNGTTVESYPT--QPQVHALIQNTNANIVRDVAVYYEVLSQD--NVLFHGNIPAFNV   259
A1285_1286_c  -----SKEMRKQSLTLAPGASKKVTGSYVKNPDIKVRTNLTFNLNYLIVKRTSNNE--PQKKGFGIKNSFAMTVPIVVTRDFDQK--ITPRLTLTKTKMDSSSG--VPQVVGQIANHEPTMFGQIKMHAWITKKNQTKKIYQKNAEKLSM   254
LSA0175       -----HFK---LQVVVPANTQKWVNVN-VKMPDTAFKGKKAFAINLAAMGPT----------GGSISNRYVYAVGVTLN-GIKLPRKELHHLQMPTMQTGFVDK--KAALQFKVNNPDPIFLKKGQLSTKLTNQKLGFFNYKLDLKKMHI   248
LSA1727_1728  -----TVP---KKTSLPAGATKRIAIK-VKT-NDGFKGTKATAINLSADQFNQKSA---------VKNAYRYAIGLILN-GQQLGKKDYRYLESPSIKTRFTKE-GKAAISVKLNNPDPVYLEKAKVKIKLQNQKWGFIQYTTSVKNGKV   255
PPE00557      -----RLP---KEVEVTPNSSQIIHGS-ISFPAKDFNGILMAGLHFTEKTTTAKKN--------TVSNTVAYNIPFVIRGNHDQR--PKPRLSLDQLKMTKPNL-STYELDAKLTNHHQNLLKSVKTVSSIKNMDNKTL--KKETKRIDI   237
LCA00104      -----SGP---EKVSVPAKTEKLVTYI-LKAPSQHFIGVISGGFYVSPENDSSTNK---SVSGVRFTNRFAIGITTILRQDFKTP--NPPLLAMTKAGIGTINH--YAAVQATISNPSGNQFGDITSDYALSKTGTKKALITGHFVGQAV   245
lp_3413       -----TVPN--EETTIPANKSRIVRAQ-LKTPTKLNKGIILGSWQVKIADQHLKTK------GATLTSEYAYNVGIKLT-SNRDP---LPKLRLADVQLKKRSG--IKTVIGNIQNYHASILGKGTVTAYVSKAGQTKKLAVADLKSSSM   250
LSA0213       -----KVHRADRVITLGPNEVKKVSAT-ITMPSEQTKGMIYGAWHFIEYGQKETDN------HSNVSGSYAYNMGIVLR-GKPYE--IYPELKYQGVNPTVRNG--HPALGIQLNNTKPMAIKNAKVKAVVYKNGFFKDKRVYETDSVDV   248
LCA00271      -----TLDKGGATIQLAPKQMKVISAT-IKMPKQRYDGMIYGDWHFIEHVDKGESG------GNAVGSNYAYSVGVVLR-GQNYE--VFPNVKYDETNPILFQK--HPALGVTLRNVEPMAISKANVSVAVGMDSGDVKTYKA--DNFMI   191
LSA0173_0174  -----KLDKKSETIKLAAQETKILKMT-VKMPEDRTNGMIYGDWHFIEYLHKKGGQ-------SSVGSNYAYSVGVALK-GQHYK--VYPELKYDKTEAMIYRR--HAAMGIKIRNTQPMVINKVSAKAVVSKEGLFSSKHVYTTSNQSV   248
LSA1729       -----AVRKSDRVLHLGPQETTVIQAT-IKMPEDNFNGLISGGWHFIEYRDNKTDE-------QTISSNYAYMISVVLR-GSHYK--VYPELKYDSTKPILYNN--RPAMGVKLRNTQPMALKNVHFKAVVSKKGLFSDKRLYEKEGSSI   251
LSA1816       -----------VKVTVPANSKKDVTIP-IKMPATTYSGIILGAMHVEKETAQQKQT----TKKAQIINHYGIEIPVVVRTTPSST--PDTKLQLKKVTAGLDTN-KHSAVLATLYNESNWVLTPLKVDAKVYRKSNHKLLYSSHKKGLEM   240
LCR01867      -----AET---TEVEIAENQSKEVLLN-LTRPSVSSPGIILGAIHVSKVPNNVEQE---ENKNQKIVNHYSAEIPVVIRTQPSST--AQLDLQLTEVTAGVTNK-NKSGILAKLANPTSWVMPHLSISTKVYQQDNNKLLYQSTDENLDM   244
PPE00547      -----SGP---QTVLLKPKEVRRVSFQ-LKIPPKGFKGTVLGGFYCYTKPVKAATS----TSGLSLTNNFAYTIGAKLE-CTSSK--IQPKLSLIKATPGLDNG--YLSIFATLQNAAPVLLSKLDMRATVTQKNHHEIL-KRTHKKISI   256
EF00339       -----KLPE--EHVKVPAKKTVTAKMT-VEIPANGFQGLVLGGVYASLAEDEKEEK----GKSTGLTSRYGFNVAIALRMSEDTPMYLAETLQLVKLVPTIALG--NKSVQAVIQNPTSAIFPEVRLEGKVTKKGETKEYAKRILPSVRF   253
EF00981       -----TLEN--EEVSVPPHQTVSAKMK-VKGLDQPIKGVVLGGVYAHLKDEKTEEE----NQTTGLTSRYGFNVAIAMRSAANVPLTEVNQLKLAKVTPTIALG--AKSLKAVIQNPYAAIFPEVRLEGQVIKKGSSKKIAQRTLKNVRF   250
OOE1273_1274  -----GHV---QRASLKAGRSKTLTFK-IKIPKKVFPERLSVDYTLKKKQIAKTIK--KAAVVFNLKNTYAVMVPVFLS-EKDKV--VHTKLLLENVKPASINK--VPVILAKISNQTPTVFGKLKITSEVLDSDGNTVISKSSNTNMQM   246
lp_2174       -----SSG---VVVKLAAHQAKTVAFK-TTIPKSGFQGEVLGGLFVTNPTANAARP--TTSQGFMLKNRYAEVTAVALWCQPQVT--IPVKLVLDEVKVTPTNG--QPVLFAKLRNLTPTLFGQLKIQARVIQRTTGKTVATQQLKSGSM   257
EF00404       -----KIKDGVKEIPLKGKEEKTITFE-ITTPESAFSGVKLGSLRFVRKNSTEVKQ----KKSGLTPQYARVIAIMLT-EDEEPFNQGAMLHLKKASLQLANG--QKVIAARIQNDQPKVLQELTIQGEIRLKGEKKILYKHKMEKFSV   254
lp_1448       -----TLAAGSDKVTVPAKSNRVVSAT-INIPKGFDTGVILGSWYFEKMGQGAKKA----SKGVNINQKYSYALAVKLT--AKEI--AQPKLTLGTVTPGLRNY--RKVVLATIRNPEPAVVSQLKLTTKVTRKGSSKVLYQNSSDNLIM   249
lp_3678       -----TIQGE-RTITIPAEGSKTVAIK-VKLPKQPVNGVLLGGWYFKRVDDKVTGV---TEKSINVKNEYSYVIGLKLS-SGKLP---ISNLCLGPVKAGLNNY--HRSIIVALRNPVARIIPNLKIRTVIINKRHQKVIIKNTKTGVMM   257
lp_2977       -----KIKGP-TTVTVPANGSKLVSAQ-TTMPTKDFNGALLGGWYFKRVDQKVTGE---VKGATNISSEYSYVVGIKYS-LGTAP--T-PVLKLSDVAAGMVNY--HRSIVVTLRNTAAVMIPRVTMKTTVTAKDSGKTVVKATKQGVML   251
lp_3066       -----KIQGP-TTITIPANGSKVVTAT-VTMPKQAVNGVILGGWYFKRVANKVTGS---VKGSMNIANQYSYVIGLKYT-AGIVP---QPQLKLDTITAGMQNY--RRGVFPQLRNVTAVIVPKLTITARLTSKNSGKVIKNFTQKNVQL   260
LCA00970      -----EAP---KTIDVGAEAQVPVTFD-VKIPEKGFTGQILGSLYVEDPTVHKSGG----SGMAINNKFAMLVGVVLQ-TSEAK--VAPELKLMDVKPGTQDN--QASVLATIQNIKPRLFGEMTLDGKVYRQGSKDPYLTRSEKGWSF   238
LX01222_1223  -----KNDS--PQLTVGGHQSHTVRFH-LTMPDQAFNGILLGGIVVCPVQEKKKVKAYKKKEGKGIQNVFIHTIALRIA-ENDTP--VHSELIGGDVKIGQENL--HNQVSMAIRNPQAKLMSQLNGDFIITKKGETKPLVNIQKQNLSI   254
LSA1810       -----DQP---QMITLKPKETKTVNFK-VKMPDQLYAGVLKGALYFKDTKAGSTQG--TDQQGFSVKNYYAMAIGVVLR-EHPNI-HTPANLKLRRIRVSETNYLHEPTVLAKLENVRAATPGAMTVNAKVSKHKGSKTLFGYQREGITM   250
EFA03601      -----LIAK--KEINVSAHAETTIPIE-VKIPAKSFKGRVLAGIHVSPKEEAETEN---ATEGAQIKNRIAYNLAVVLQ-ESQET--IEPDLKLLSGDLDEVNA--KPTVQLRFQNPQPRIISNLIFTSKIFYEN--QLYIENTSNEFLV   246
LSA0610       -----KPAK--KNITLTAHEQKVVNIR-LSAPDKAFKGQLVGGISATEVPTTAKTT-----QQISVRNKFAYAVAVVAR-SNDQS--VTPEFKLGAVKTGQHMG--TNQIIANIRNVQPKFQNQTSLNAFVTKRNQKTKLYQTEKKQVQF   247
lp_3072       -----TTAA--NKITLAKGTSKIVTFK-VKMPAQKYDGVVVGGLSFLKKAAKQQSK-----SAMAIKNQYAYSVAVVLH-GDHAL--TKNKLTLGKIDATQNNG--YNQISLPLQNRTAAFLNQVKTDVQIYRRGSNKVRYKQVNEHGQM   246
lp_3115       -----TTDE--PQVKLAKGQVKTVKFQ-IKMPAQSYQGILAGGITFLKKATATDTK-----KAVAVNNQYAYTEAVILH-GSKDL--TTNQLTMNKITVKQLNG--RNAINFPLVNHTAAYLNKVQTNIKIYHRGGSQVVYHQKLANGQM   245
LIN00803      -----TSDL--PIVEVAANSEQIVSLK-LKIPEESFKGVLLGGITIEPFQDETKQE--------QVNNVYTRTFAIQIS-ENDEP--IPAKLEAGEVSLSQINY--HNVVSLLIKNKAPTIITSVEANIKVTKKGQTEPLLNEVKKDMSI   242
LSA1818_1819  -----SPA---QRLKVPAESSRTATFT-LNAPKATFEGTILGGFYVSQVKQDQVEQALDHKKGTSLTNRYSYILGVKLT-QSDQT--VKPHLRLNRIKPGLLNG--HTAILSNIQNTKANSVGEMQVDAKIRRAGQSKVLYHSKQTDLQM   252
FA03978_3983  -----KTV---GEITVPPQSAVTETLS-LKAPEKELAGVVAGGITFQEKKENVSNK-----STVGVVNQFSQVKAILIX-NSEKE--SSPDLKLKKAYADQSDG--RNVIKANLQNPNASYMFGLTTSVEIYKKETKEPYLSIKKEKIDV   248
lp_3451       -----SKS---QVVTVPAKSSKKVTIK-LTVPEKKFAGVALGGINIIQELSTKAKQ---SSSGMAINNQYAYVIGLQLRESDPSG--IKPVMKLHAVKAKQRNY--RNYVTANLQNNQPVIMHGLKIKSYVTKAGSSKKLLTTTKENMSM   244
CR01206_1207  -----TSP---RIVTLAPLTRQKIDIK-VKMPNESFDGQIAGGITFSKEGQNDKKE---DSEGITVKNTYSYTIALLMK-QNENE--VSPDLKLTKISPGQING--RNVINVNLQNPTMAYINTMNVKATIKGITDSDIAYYYSNPMMQM   243
LX01386_1385  -----NTP---KVVTLPPLTRQKLDLK-VKMPNEAFDGQIAGGITFSKEGQNDKEE---DSEGITVKNTYSYTIALLMR-QNDNE--VSPDLKLKTVSPSQING--RNVINVNLQNPTMTYINTMNVKATISGISNTDIKYTYSNSMMQM   243
EF02289       -----QLKDGGESVELPAKSEKAVELR-VKMPKEEFSGQLAGGITFSEKVDETKDKQKENTNGLAIENRYAYTVAVLLR-ENETV--VQPELSLEKVEPTQRNA--RSVISATLLNHEAAYLQSMKVTANVKNKKTNNVILEKEQEDMQM   248
LCR00179      -----NIPK--KEITIAAHAQATLAAK-VSMPKGMDRGSLAGGFSFKEKESNQKSN---SSDGISIKNEYQYVIALVTQ-QNTQK--VSPQLKINEVKATQVNS--RNVFAANLQNISPTYLRDMNATATVKGVTNSELKYSFENSDMKM   252
LCR00709      -----EIPK--KEITISAHSQVTVTAK-IKMPQGANSGVLAGGFAFKEKKSKNQES---TSKGVSITNEYRYVIALVIQ-QDTQK--VSPQLKMNDAKATQVNS--RNVFTANLQNMSPTYLQDMNAKATVKNVNNPEIKYSFENSDMKM   253
LCA00297      -----KTP---AQVKIPAKGSTVLNLD-VTMPSKPLHGVMAGGITLKEHQPGDQAA-KDAGKGVAINNRFSYVIGLLMQ-QSTDQ--VAAHVKLNDVKPSQINY--RNVILASMENDAATFVNKVAVDAKIMAKGSDKSVYQVAKEGLQM   244
LME00909      -----KGP---SSVVIPASSSKNVAFH-ITMPKESFDGILLGGLTFQQKSSEVNQD--KAKNGTTVQNEYSYAVAILLK-ETDKV--VDPNLNLLQVNPGQINY--RNVINAELQNDQSVLMSKVSVDAKIYSKNGKKPVYTSIKSDMQI   248
LCR00188      -----SIP---KEVSLQPNSSQQVKVS-VTMPKEIFNGVIAGGITFKEKDSEKINS---NSKGLSIQNKYAYVVALLMQ-QNKNT--VAPDLKLNKVEPSQVNY--KNVINANLQNPMAGYLNQMYVQAEVKGVSNSKLSYKANKEMLQM   243
LX01614_1615  -----SIP---KEVSLQPNSSQXVKVS-VTMPKENFNGVIAGGITFKEKDSEKTNS---NSKGLSIQNKYAYVVALLMQ-QNKNT--VAPDLKLNSVEPSQVNY--RNVINANLQNPMAGYLNQMYVQAEVKGLSNSKLSYKANKEMLQM   243
EF02664       -----EAP---KEIILPKHSQKTLPLT-ITMPKDSFDGVMAGGITLKEKKKETTTS-ADQSKGLAINNEYSYVVAIILQ-QNETK--VQPDLKLLGVKPGQVNA--RNVINVSLQNPQAAYLNQLHLINTVSKGG--ETLYQSDTEDMQV   243
EF01003       -----QYP---SSVRLKPKSEQTVSIN-VKMPNTPFDGVLASGITFKEETSDEGKRQDDKSQGLSIKNEYSYVVALLMQ-QNKKK--VEPNLLLKKVSPGQINA--RNVILVNLQNDQKTYINQVAFSAEITKKGHEEVLYKEEKANMQI   248
EFA04468      -----SYP---QIVTLKPKSVQTVAFH-VRMPNERFDGVLAGGITFQEIQTEKDQT-ETKDQSLSIENEYTYIIALLMQ-QTLNE--VAPNLTLHEVKPDQINA--RNVILANVQNDQKTYINQVVIETKITKKGHSEVLYQEEKEGLQI   246
EF01894       -----QLP---KEIHLPKHSTKTVECQ-LTLPEKPFNGYVLGGLYFEQKSDEQPAH---SENGVAINNRFSYVVGVLLS-ETDEP--VQPELSLNEVKTDQANG--RNRVLMNLQNKQAAMIKKLQVDASLYYEKETKPRYENHQESLTM   246
LIN00594      -----TIPDDQKLLTIPVNGKKTVQVT-IEMPDESIDGVVLGAVQFKKKNQTETKK----TKGVSLKNEYSYIVGMQLS-ETDKQ--VKPHMNLLSIKPTLINY--HTAIVAKLQNDQPVIIKNLSIDAKVYQDGSNKMLYQTKKADMTM   249
LMO00586      -----EIPDDQKRLTIPVNGKKTVQVM-IEMPAEKVDGVVLGAVEFKKKNTNETKK----TKGVSLKNEYSYIVGMQLS-ETDKQ--VKPHMNLLSIKPALINY--HTAIVAKLQNDQPVILENLSIDAKAYQQNSNKLLYQTKKTDMKM   249
LIN00560      -----KASE--SEVTLKPNETKIWSLD-INMPAESYDGIILGGLHFQEKKDEEKEK-DTSKKEVQIKNEYAYVIGVKLT-EKTTI--VKPNLELNEIKPASRNY--RNVVEINLQNTKATLVGGMAVDAKIYKDGSKKVLRESKRTDLSM   247
LIN00555      -----EVSD--SEITLEPNETKIWSVN-IQMPEESYDGIILGGLHFKEKTKEKTEKETKQEKEVQIKNEYAYVIGVKLT-EKTTI--VKPELALNQIKAGSRNY--RNVVEVNLQNTKATLVGGLAVDAKIYKQGSEKVLHEAKRTDLSM   247
LMO00552      -----KESV--SEVTLKPNETKIWTAA-IQMPAENYDGIILGGLHFQEKKEEDKEK-ESSENDVQIKNEYAYVIGVKLT-EKTTV--VKPELELNQIKPATRNY--RNVVEMNLQNTKATLVGGLAVDAKIYKKGSEKVLHESKRTDLSM   246
BTH03757      -----KMP---KETKVPANSIVTVDIN-LEMPSESFDGVILGGLYFKEKEDENAKK---KDEGVQIENKYAYAIGVVLK-ETDAE--VKPDMKLNEVKPTQING--RNVVTANLQNVKPAMLKNLSVDAKVYKEKGTDVLFEARKENL--   240
BCU00754      -----KVQ---KETKIPAKSTITLDVN-IEMPNESFDGVILGGLYFKEKENDKEKK---SAKEVQIENKYAYAIGVVLR-ETDVE--VKPDMKLNEVKPDQVNA--RNVVTANLQNIKPAMLKNLSVDAKVYTETGKDVLHETKKENLRM   245
```

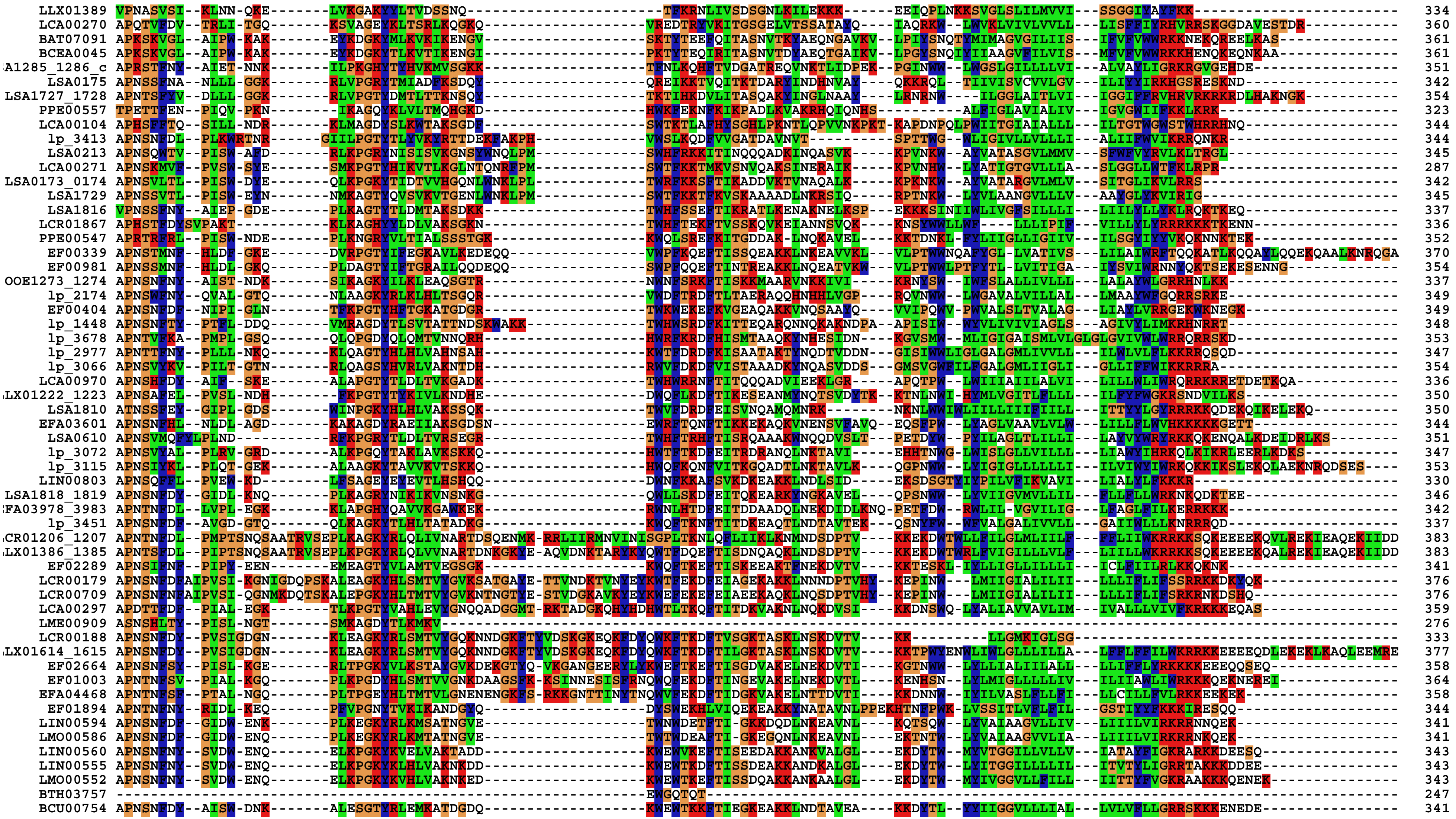
LLX01389 334
LCA00270 360
BAT07091 361
BCEA0045 361
A1285_1286_c 351
LSA0175 342
LSA1727_1728 354
PPE00557 323
LCA00104 344
lp_3413 344
LSA0213 345
LCA00271 287
LSA0173_0174 342
LSA1729 345
LSA1816 337
LCR01867 336
PPE00547 352
EF00339 370
EF00981 354
OOE1273_1274 337
lp_2174 349
EF00404 349
lp_1448 348
lp_3678 353
lp_2977 347
lp_3066 354
LCA00970 336
LX01222_1223 350
LSA1810 350
EFA03601 344
LSA0610 351
lp_3072 347
lp_3115 353
LIN00803 330
LSA1818_1819 346
FA03978_3983 342
lp_3451 337
CR01206_1207 383
LX01386_1385 383
EF02289 341
LCR00179 376
LCR00709 376
LCA00297 359
LME00909 276
LCR00188 333
LX01614_1615 377
EF02664 358
EF01003 364
EFA04468 358
EF01894 344
LIN00594 341
LMO00586 341
LIN00560 343
LIN00555 343
LMO00552 343
BTH03757 247
BCU00754 341

```
      LLX01389 -------------   334
      LCA00270 -------------   360
      BAT07091 -------------   361
      BCEA0045 -------------   361
 SA1285_1286_c -------------   351
       LSA0175 -------------   342
  LSA1727_1728 -------------   354
     PPE00557 -------------    323
      LCA00104 -------------   344
       lp_3413 -------------   344
       LSA0213 -------------   345
      LCA00271 -------------   287
  LSA0173_0174 -------------   342
       LSA1729 -------------   345
       LSA1816 -------------   337
      LCR01867 -------------   336
      PPE00547 -------------   352
       EF00339 RDDDNEK------   377
       EF00981 -------------   354
  OOE1273_1274 -------------   337
       lp_2174 -------------   349
       EF00404 -------------   349
       lp_1448 -------------   348
       lp_3678 -------------   353
       lp_2977 -------------   347
       lp_3066 -------------   354
      LCA00970 -------------   336
LLX01222_1223 -------------    350
       LSA1810 -------------   350
      EFA03601 -------------   344
       LSA0610 -------------   351
       lp_3072 -------------   347
       lp_3115 -------------   353
      LIN00803 -------------   330
  LSA1818_1819 -------------   346
 EFA03978_3983 -------------   342
       lp_3451 -------------   337
 LCR01206_1207 LNKK---------   387
 LLX01386_1385 LNKK---------   387
       EF02289 -------------   341
      LCR00179 -------------   376
      LCR00709 -------------   376
      LCA00297 -------------   359
      LME00909 -------------   276
      LCR00188 -------------   333
 LLX01614_1615 QISKEDNSDETDV   390
       EF02664 -------------   358
       EF01003 -------------   364
      EFA04468 -------------   358
       EF01894 -------------   344
      LIN00594 -------------   341
      LMO00586 -------------   341
      LIN00560 -------------   343
      LIN00555 -------------   343
      LMO00552 -------------   343
      BTH03757 -------------   247
      BCU00754 -------------   341
```

Figure 4:
Multiple sequence alignment of CscA proteins. Proteins IDs can be found in additional file Table 5. Colour coding of highly conserved residues: green, hydrophobic (L, M, I, V); blue, aromatic (W, F. Y); red, basic (R, K, H); light brown, neutral and small (P, G, S, T).
