# Supplementary material for: Lactobacillus plantarum gene clusters encoding putative cell-surface protein complexes for carbohydrate utilization are conserved in specific gram-positive bacteria
Source: BMC Genomics. 2006 May 24;7:126. doi: 10.1186/1471-2164-7-126 (PMC1534035; doi:10.1186/1471-2164-7-126)
Supplement: Additional file 7 — Figure 5: Multiple sequence alignment of CscB proteins. [file 1471-2164-7-126-S7.pdf]

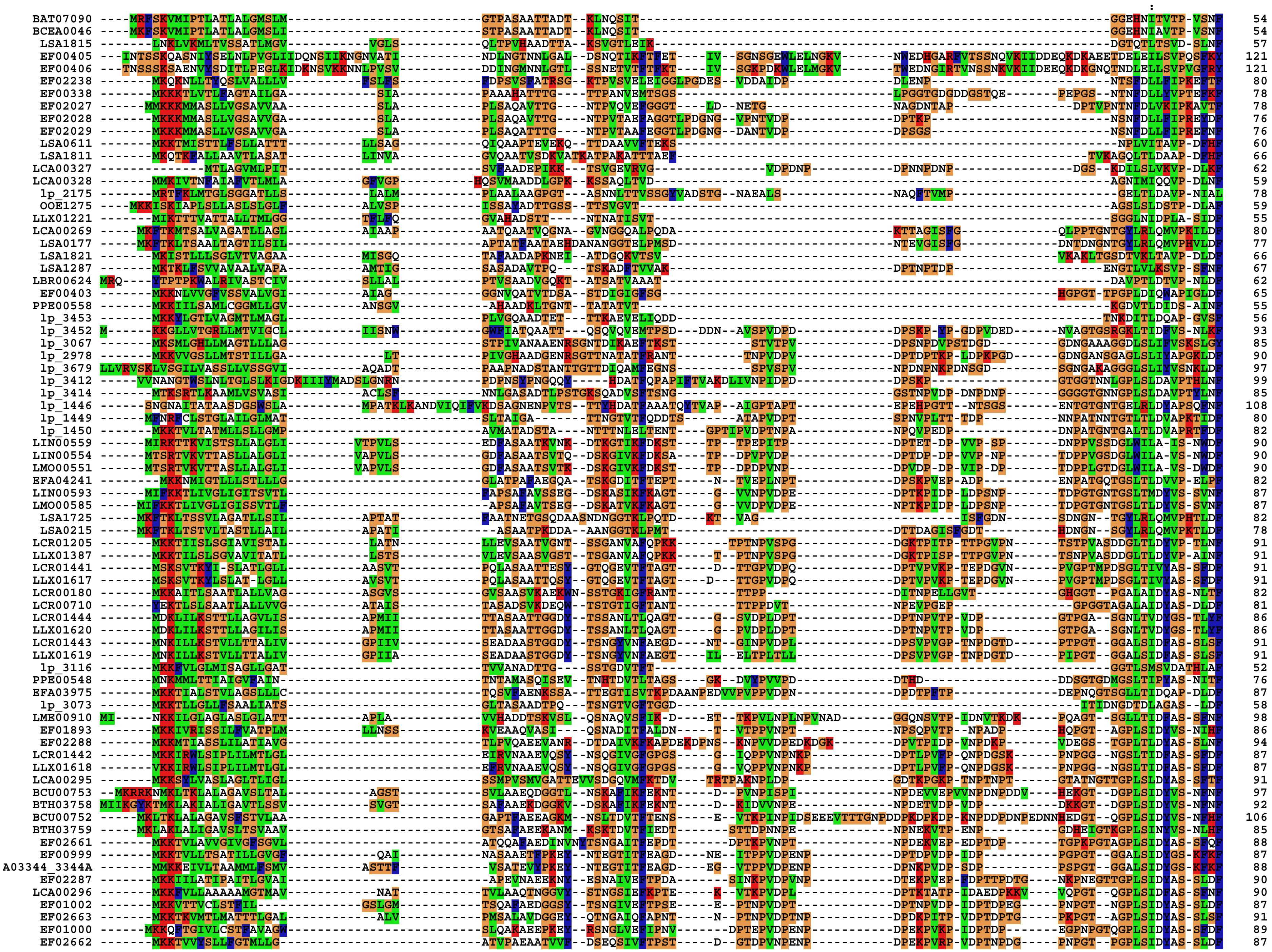
BAT07090 54
BCEA0046 54
LSA1815 57
EF00405 121
EF00406 121
EF02238 80
EF00338 78
EF02027 78
EF02028 76
EF02029 76
LSA0611 60
LSA1811 66
LCA00327 62
LCA00328 59
lp_2175 78
OOE1275 59
LLX01221 55
LCA00269 80
LSA0177 77
LSA1821 66
LSA1287 67
LBR00624 62
EF00403 65
PPE00558 55
lp_3453 56
lp_3452 93
lp_3067 85
lp_2978 90
lp_3679 97
lp_3412 99
lp_3414 85
lp_1446 108
lp_1449 80
lp_1450 82
LIN00559 90
LIN00554 90
LMO00551 90
EFA04241 82
LIN00593 87
LMO00585 87
LSA1725 82
LSA0215 78
LCR01205 91
LLX01387 91
LCR01441 91
LLX01617 91
LCR00180 82
LCR00710 81
LCR01444 86
LLX01620 86
LCR01443 91
LLX01619 91
lp_3116 52
PPE00548 76
EFA03975 87
lp_3073 58
LME00910 98
EF01893 86
EF02288 94
LCR01442 87
LLX01618 87
LCA00295 91
BCU00753 97
BTH03758 92
BCU00752 106
BTH03759 85
EF02661 88
EF00999 87
A03344_3344A 88
EF02287 90
LCA00296 90
EF01002 87
EF02663 91
EF01000 89
EF02662 87

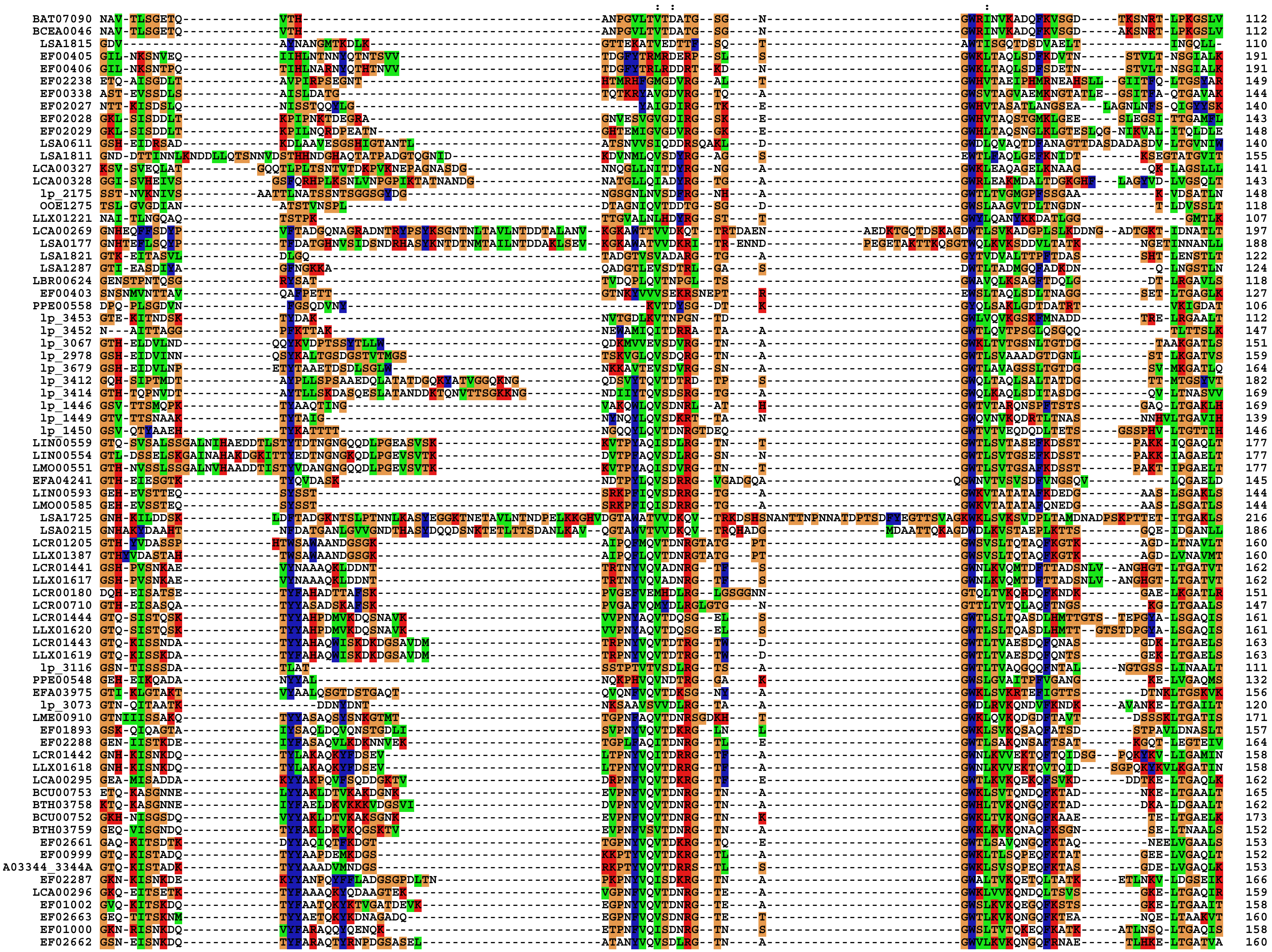

BAT07090 112
BCEA0046 112
LSA1815 110
EF00405 191
EF00406 191
EF02238 149
EF00338 144
EF02027 140
EF02028 143
EF02029 148
LSA0611 140
LSA1811 155
LCA00327 141
LCA00328 143
lp_2175 148
OOE1275 118
LLX01221 107
LCA00269 197
LSA0177 188
LSA1821 122
LSA1287 124
LBR00624 118
EF00403 127
PPE00558 106
lp_3453 112
lp_3452 147
lp_3067 151
lp_2978 159
lp_3679 164
lp_3412 182
lp_3414 169
lp_1446 169
lp_1449 139
lp_1450 146
LIN00559 177
LIN00554 177
LMO00551 177
EFA04241 145
LIN00593 144
LMO00585 144
LSA1725 216
LSA0215 186
LCR01205 160
LLX01387 160
LCR01441 162
LLX01617 162
LCR00180 151
LCR00710 147
LCR01444 161
LLX01620 161
LCR01443 163
LLX01619 163
lp_3116 111
PPE00548 132
EFA03975 156
lp_3073 120
LME00910 171
EF01893 157
EF02288 164
LCR01442 158
LLX01618 158
LCA00295 162
BCU00753 165
BTH03758 162
BCU00752 173
BTH03759 152
EF02661 153
EF00999 152
A03344_3344A 153
EF02287 166
LCA00296 159
EF01002 158
EF02663 160
EF01000 158
EF02662 160

```
BAT07090     LNSGGA-----TIT--KVGG---------------TSSPN--------------------------LEFKTTNFIID-------TDNQV--TILSAK----------KDEGMGKYNVS------------------------FNDKSLA   170
BCEA0046     LNSGGA-----AIT--KVGG---------------TSSPN--------------------------PEFKTTNFIID-------TDNQV--TILSAK----------KDEGMGKYNVS------------------------FNDKSLA   170
LSA1815      ----------------SATP---------------K-------------------------------------------------------AVFTKT----------ADAKFGEQTAA------------------------VKDANMS   139
EF00405      MENMKIESIKNRDSPQESID---------------QNPTG--------------------------TPSTVSTNETL-------ISGQAAKTLINAQ----------ANEGHGTWQLR------------------------IPFDKVS   258
EF00406      MENMKIESIKNRDTPQESID---------------QNPTG--------------------------TPSTVSTNETL-------ISGQAAKTLINAQ----------ANEGHGTWQLR------------------------IPFDKVS   258
EF02238      YDKTLRRF-FMTNTMYGLDF---------------SEDP---------------------------AAPDFPTNPII-------IGNGA-TLMSNAG----------DRKGQGMWSGR------------------------MTDISLA   213
EF00338      YDETAKTY---SRD--VAAF---------------AADP---------------------------GSPEFAGTTIP-------VGGAA-VSIATAA----------VGKGQGTWDSE------------------------LSNVKLN   204
EF02027      FDATKQIYVYLTDAESLANY---------------EADP---------------------------TTPDFSGTNIP-------LSGDA-IAIATAS----------SGKGQGIWYSG------------------------IRTMNLE   205
EF02028      YPLIYNEETKTYDY--QSAD---------------LADPVTKP-----------------------ETVDTTAFSIA-------LGGDA-VLLANAT----------AGKGQGLWQFSF-------------------------RPYLN   209
EF02029      YEATSNSYVALTTA--VSTP---------------EVRP---------------------------QLAAPQNWTLD-------LGGEA-QLMANAA----------AGQGQGLWQFS------------------------LFNTALN   211
LSA0611      FKTPNV-------T--IDGA---------------NKGDA--------------------------ALPTTSDQTVQ-------VNDQA--STFMKG----------GATSFGSVAATLGTAPTSG----------------TDSDAIF   204
LSA1811      IGKGVD----------NLTN----------------------------------------------TTIGKDAQVIM----------------NTAKTLKNTP----------EKLGAQTTTADFSA---------------------PGDKASS   207
LCA00327     AGVAKTD---------ETDN----------------------------------------------NPTAISQAELV-------LEGSG-VQVWNAA----------SGQGQGRNTGV------------------------FTSADTV   193
LCA00328     ASNPDI------------NP---------------INPAG--------------------------SNVPRLNFAKD-------NTSVSSTVVWQAD----AETGTKSGQGGQGNNQTTI----------------------GTRTALT   205
lp_2175      LNMTKG------NA--DNAN---------------TAAPT--------------------------ALSLNQSEVTN-------GWVASPQTLWNAA----------ADSGEGSNTA-------------------------TTATSSS   206
OOE1275      INNVSI-------------------------------TPS--------------------------------------------TDGSTTSTVWDNS----------ASTGGTLTQA-------------------------LSSSNTS   157
LLX01221     LAPTS-------------------------------------------------------------DQGKVAAVSLN-------GSAQN-IDVLDKA----------DATKPDI----------------------------KITLSPS   149
LCA00269     MLNTAYGQTGNVYG--LTNE---------------SQDDGFTPVGAL--VPLT-------------DISKTTTMTLS-------GTDTN-HQVAHAA----------TGEGEGANVFG------------------------WDKTNIK   272
LSA0177      LKNTAYGRTADVFTLTNDAQ-----------DSDFQADPTIDDKDVS-------------------TITNNVSLSLD-------GKGEE-VLVADAA----------DSEGQGANVFG------------------------WDHSDIN   265
LSA1821      LKNASG------IS--TDNA---------------AH-----------------------------TVADTKSVDLT-------NDSAK--NIITAG----------KDQGLGNWNYK------------------------ISGSNLH   176
LSA1287      LASTGS----------LGED---------------------------------------------------LATAIK-------DDASA-VEVVKGN----------GAHGVDTFNMA------------------------AADSKLT   170
LBR00624     LGAPAI----------MSLN---------------TSNSS--------------------------TPPTANQVNVD-------GSGSD-QVIWSAP----------QNGGLGQWSAT------------------------YAANTVQ   174
EF00403      FDAAVK-----GYT--GTNT---------------PESPGSIVDKGART-----------------ATVNASSQSVT-------AGAAA-VPVMKDA-----------GTGGASFLGSTA----------------------MEMSNVK   196
PPE00558     LSDAAA--------------------------------------------------------------------------------------VVASK----------DSNIVG-----------------------------ENADKLS   130
lp_3453      FAQVNA----------TADD---------------AN-----------------------------NISKAKAYKVD-------ITDQN-QIIMDAE----------ANEGIGKFTSK------------------------YATNEVS   165
lp_3452      LGAVQL-----RPG--AGNV---------------STAPN-----------------------------VVNTGELV-------AGLAN--NVVIAP----------PNTGLGTWLVVLN----------------------RGQVPTE   204
lp_3067      LPKGDV----------VNSG---------------SEANG--------------------------AVATASEVALN-------GTVGS-SAVLNAD----------KGGGSGVTVS-------------------------RDPKALT   206
lp_2978      LPAGTVISSGSEASNGSTGG---------------NGAVGQAVELDL-------------------GSQASTSAGAS-------FTGTTSGNVMTAA----------DKSGAGITSDL------------------------LDPAAVK   233
lp_3679      LPKGTV----------TNSG---------------ATNN---------------------------GVQSTEALNVL-------DGNSA--TVLSAA----------KDHGAGVTVDQ------------------------IDPSNVK   218
lp_3412      LTSGTAQYLNASTSKWVTAT---------------DQN----------------------------QATLPAVIKLT-------PGATQQTLIAGTT----------SQQGVGTNQQI------------------------WNVNNVA   247
lp_3414      LSGGTPQYLTGEANQQSWVT---------------AADAN--------------------------QATVATPIVLN-------AGASSTTDVATAT----AGSKVGDGQGMGVNQQY------------------------WNINNVQ   242
lp_1446      IPAGQT----------YNER----------------------------------------------ATGQLKSYAVD-------LTTAE-QPVFAAP----------ATANVGKDLSTNV----------------------WQPTQVQ   222
lp_1449      LPQGTA-----RNS--LNEP---------------ASVA---------------------------DSNLVVAPSVS-------VTTAD-QVVFSAP----------NRAGVGKATSTNT----------------------WNAGAVT   199
lp_1450      VPAGIA----------RNSL---------------ATDPTAVD-----------------------TNLTTTAADVT-------NTSGA-VTVFNTT----------ATTGAGKGTSVKV----------------------WDPSDVT   207
LIN00559     IPKSTV----------ATVN---------------STAK---------------------------APTGYDNITIP-------MTGLSTVPVMAAKDKQTATPTNFSDDEGMGTWTDSFGSAAKNA---------------TQTTKPK   252
LIN00554     IPKSTV------KS--KDST---------------AKAP---------------------------TGYDNVAISMT-------GGAAV--PVMAAKDLQATTPTDFSDDEGMGTWTDSFGSAEVSA---------------TETSKPK   252
LMO00551     IPKSTV----------SSAT---------------STAQ---------------------------APTGYDSVTIS-------MTGGAAVPVMAAKDMQTATPTNFNDDQGMGTWTDSFGSQAVSA---------------TDTSKPK   252
EFA04241     FGTSTV------KSTSDNES----------------------------------------------TGPTSQTVTGL-------SKASAATPIFTAA----------KDQGLGTWLSV------------------------YDPANIT   201
LIN00593     FKNGET----------VSSS---------------TTAT---------------------------TPTAVQTVELP-------TDGTSIVNVVSAK----------ESEGMGTWVNRWFGATPNDAA--------------SLNDNVK   210
LMO00585     FKNGET----------ASAS---------------TTAT---------------------------TPTAAQTVELP-------TDGTSIVSVVSAK----------ESEGMGTWINRWFGATPNDTA--------------SLNDNVK   210
LSA1725      FDNTQYGQTQQVAELTKDTQDATYTTDLA------AVTPAPKVAKVS-------------------SITNNFSTPLA-------AGGTD-VTVAEAK----------DGEGQGANVFG------------------------WTPENIK   298
LSA0215      FKHTKYALTSGVYELTNSDQDKTFAKDLADYKASIATPPATPKADGDDGDPAPAPSAGDVAINETAKDAITDTFSLSLAAPTSKLGTAAGTVVGKAD----------TDTGTGANVFA------------------------WAPEDIQ   301
LCR01205     FNTDKT-----NNV--VSTS---------------TSKAS--------------------------STVNTATNPLT-------PGTAV--NIFQED----------AGTGTGTNLLSFGAADGSSLVTDSESLDASTNTTGKVDPNIT   242
LLX01387     FNNDKT-----NTV--VSTS---------------MNKAS--------------------------STVNTATNALV-------PGTAV--NIFQAD----------AGTGTGTNLLSFGAGDGSTLVTDPETLDSATNTTGKVDPNIT   242
LCR01441     LGDAEI----------QGAG---------------TANP---------------------------ADISSAITVLT-------PGVQS-TTILGAT----------AGHGSGNSLLDFGGKDGA-----------------KKDTAIK   224
LLX01617     LGDAEI----------QGAG---------------TANP---------------------------ADISSATTVLT-------PGVQS-ATILGAT----------AGHGSGNSLLDFGGKDGS-----------------TKDTAIK   224
LCR00180     FSSGKA------AN--AGGG---------------TYTPT-----------------------------AKGSFTLK-------LDTAQ--PVMS------------TDGTVGQFLTSYGKATDYREN--------------NAGGPIS   213
LCR00710     FSSGAG-----VNA--DASG---------------SYVPK-----------------------------STASYTLT-------PGTAQ--EVMS------------TDGTVGQYLTSYGKAADYQGN--------------EAGGPIS   210
LCR01444     FTGGTL----------LGGN---------------GITAG--------------------------KPSDVVASGAL-------TPGVA-TKLVSAA----------KNEGAGTWLYNFGDVDAYDASSVDTANTTDHTKV-ATKSAIS   240
LLX01620     FTGGTL----------LGGN---------------GITAG--------------------------KPSDVVASGTL-------TPGVA-TKLVSAA----------KNEGAGTWLYNFGDVDAYDASSVDTANTTDHTKV-ATKSAIS   240
LCR01443     FSKGYT----------DGTT---------------TAKPG-----------------------------YINTTNFV-------VSTAA-SKILGAG----------PNQGMGTWVYGLGANADYQENGGAHLGNEAV----STASPIS   236
LLX01619     FSKGYT----------DGTT---------------AAKPG-----------------------------YINNTNFV-------VSTAA-SKILGAG----------PNQGMGTWVYGLGANADYQENGGAHLGNEAV----STASPIT   236
lp_3116      VAGTES-----------------------------DSAVT--------------------------NGSTTLTPGTT-------SAAGAAGTVASAK----------NGTGNGAST--------------------------VTFSDSK   162
PPE00548     LSNGKV----------VTKN---------------NKSAM--------------------------PKITQDSFKLN----------------RAYQVVMTAK----------EGEGAGAFAAVFEGKN-------------------GNNENVK   191
EFA03975     FKNAVI-----RNS--ATNS---------------SGMPS--------------------------TVSGTGAEGIE-------IPADQAVDLVTAG----------ANEGIGTWIYSLGENVE------------------QGRQSVE   222
lp_3073      LNGVLD------SNASTTGA----------------------------------------------DATANTGVALD-------TLNSD-KVLMTAA----------VGKGNGKNV--------------------------ADIAKSQ   173
LME00910     ISNGKA----------VNGN---------------GSDK---------------------------VINTVDTNSIT-------IGREN-TTVMSAD----------DGEGIGTWIYSMGNQN-------------------TADKSVS   231
EF01893      FLAATP----------NSTQ---------------LLS----------------------------LAPLTVPVTLD-------PTGAATSPVATAA----------LSTGMGTWTLAFGSGA-------------------TAAQGIQ   217
EF02288      FQNASA----------VTAG---------------QSA----------------------------APTSLASVTFG-------TVGES-HNVMVAA----------TDQGAGKWSYLYGNASSIAEVDGR-----------QVMKDVQ   231
LCR01442     LKSSET------AS--FTNE----------------------------------------------KSPKTQTISGL-------IPGVE-TQVTLAS----------TGEGAGTWVIRWGNDSMVTED--------------GLTTAVS   221
LLX01618     LKSSET------AS--FTNE----------------------------------------------KSPKTQTISGL-------IPGVE-TQVALAS----------TGEGAGTWVIRWGNDSMVTED--------------GLTTAVS   221
LCA00295     FLNGNV------TS--TIDQ---------------QYAPT-----------------------------GQKEITLV-------PGSAEVAPLAAAK-----------DQGMGTWIYRFGDEQ-------------------TMAKSIE   222
BCU00753     LKNGTP----------NSAS---------------GSD----------------------------APAAAQDITLV-------PGQAS--DVLTAQ----------EEQGMGTWTNAFGKSVE------------------EGKKSVE   224
BTH03758     LKTSAL----------KSNA---------------GSD----------------------------APTAEQSITLN-------PSGAA-SDLIDAK----------ENQGMGTWVNTFGKDAT------------------EAKQSVE   222
BCU00752     LSNPFV------NS--ATDK---------------KFSPT------------------------------AKEVTLN-------PDGST-QEVTIAE----------AGNGMGTWTTAYGKDNV------------------EGAKSVG   233
BTH03759     LSNPAV------NS--VTDA---------------QYAPT----------------------------TFKNKVTLA-------PGGDA-VEITTAA----------KNKGMGDWTTAFGKGTE------------------QGKQSVS   214
EF02661      IANAGV------TS--IVDA---------------AYAPT-----------------------------PTAAHTFV-------PGTEV--ELVKAE----------DGKGMGTWVYRFGKDAT------------------EGATAVK   213
EF00999      FTKAEA----------ASMV---------------DEKYK--------------------------PTEVSSTISLT-------PGVNN-NLAMNAK----------KETGVGTWVYRFGSNEA------------------TNKEAVQ   214
A03344_3344A FTKGQA----------VSLV---------------DPTYT--------------------------PQTVNSELTLT-------PGGNN-TLAINAK----------SGTGVGTWVYRFGANEN------------------ENQDAVQ   215
EF02287      LSQNKA----------VSNS----------------------------------------------KAVTPTAHEVT-------LVAGDSSNVMSAA----------EGQGSGTWLSAFGGIEKVEVDGESV----------DKNTAIT   232
LCA00296     LKNGHV----------VTAS---------------TAA----------------------------HPDGTAEMALV-------PGAEQ--TVMNAK----------TGSGTGTHLLNWGKDAD------------------DAARSVE   218
EF01002      FKNGNV----------VTAS---------------DSG----------------------------KPTGPATITLN-------SDGSQ-SDVMSAA----------KGNGAGTYLFDWGTDAT------------------TAAKSIE   218
EF02663      LSNGRV----------VSAS---------------QSAK---------------------------PTTAPATIELN-------PTGAE-SVVMAAG----------DKEGAGTYLMSWGDSVD------------------TAKTSIS   221
EF01000      LANPTV------NS--NAQN---------------VVKPE-----------------------------ATNKIALV-------PGTAS--LVAAAK----------QGTGAGTWATYWGKVEVVAERDETNTVHNV-----NVTKDVA   231
EF02662      FTEPSV----------RSNA---------------TDVL---------------------------PPTATANIQLD-------AAGAE-TVVMQAP----------EKTGAGTWITLWGQAEKVTEKNQQGQQVNA----TITRAIS   234
```

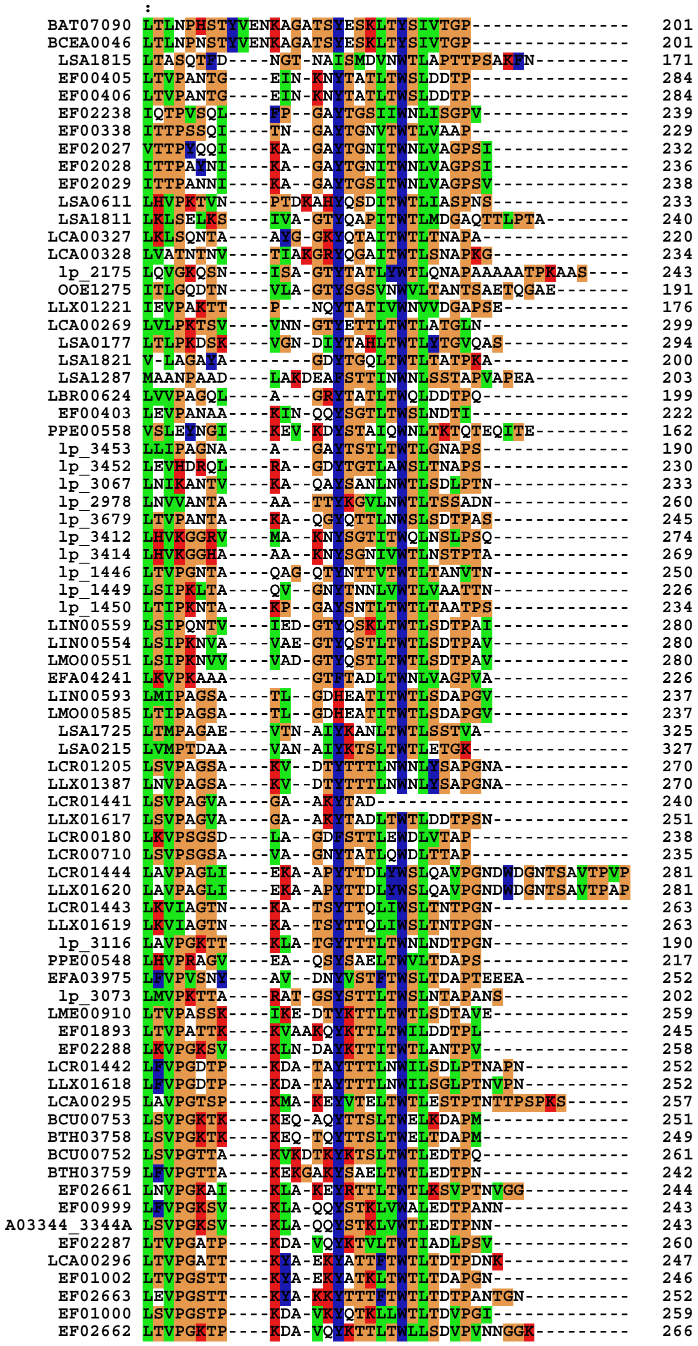

Figure 5:
Multiple sequence alignment of CscB proteins. Proteins IDs can be found in additional file Table 5. Colour coding of highly conserved residues: green, hydrophobic (L, M, I, V); blue, aromatic (W, F. Y); red, basic (R, K, H); light brown, neutral and small (P, G, S, T).
