# Supplementary material for: Lactobacillus plantarum gene clusters encoding putative cell-surface protein complexes for carbohydrate utilization are conserved in specific gram-positive bacteria
Source: BMC Genomics. 2006 May 24;7:126. doi: 10.1186/1471-2164-7-126 (PMC1534035; doi:10.1186/1471-2164-7-126)
Supplement: Additional file 8 — Figure 6: Multiple sequence alignment of CscC proteins. [file 1471-2164-7-126-S8.pdf]

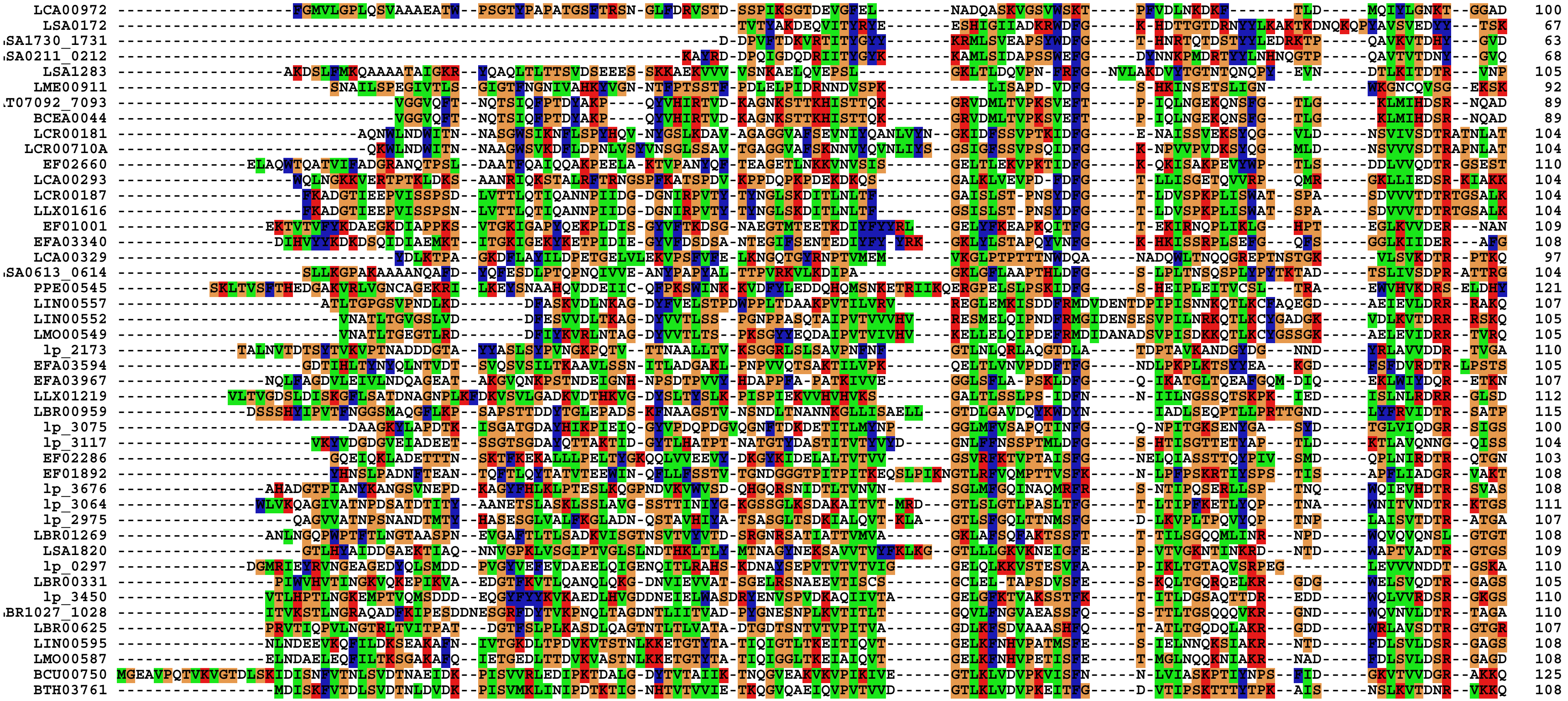
LCA00972 100
LSA0172 67
SA1730_1731 63
SA0211_0212 68
LSA1283 105
LME00911 92
T07092_7093 89
BCEA0044 89
LCR00181 104
LCR00710A 104
EF02660 110
LCA00293 104
LCR00187 104
LLX01616 104
EF01001 109
EFA03340 108
LCA00329 97
SA0613_0614 104
PPE00545 121
LIN00557 107
LIN00552 105
LMO00549 105
lp_2173 110
EFA03594 105
EFA03967 107
LLX01219 112
LBR00959 115
lp_3075 100
lp_3117 104
EF02286 103
EF01892 108
lp_3676 108
lp_3064 111
lp_2975 107
LBR01269 108
LSA1820 109
lp_0297 110
LBR00331 105
lp_3450 110
BR1027_1028 110
LBR00625 107
LIN00595 108
LMO00587 108
BCU00750 125
BTH03761 108

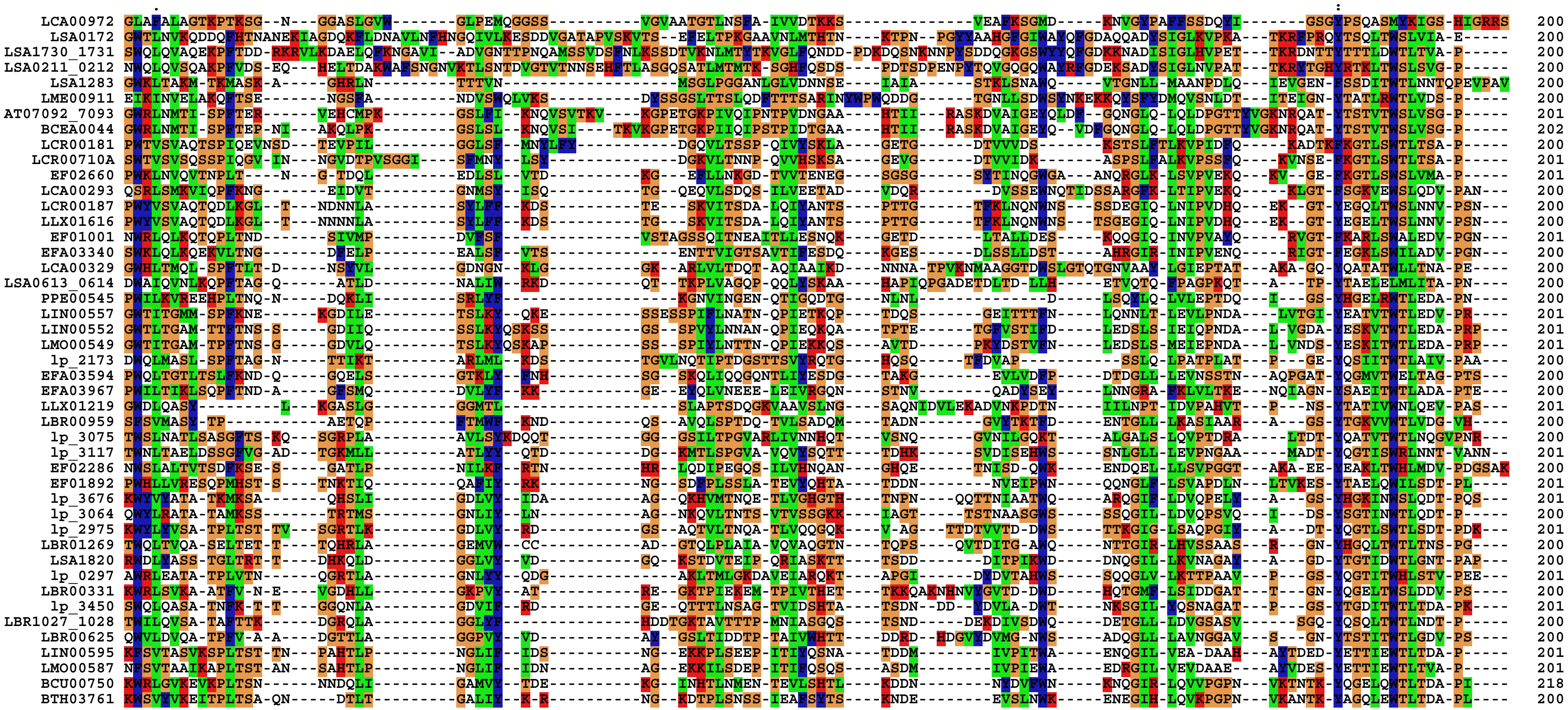

Figure 6:
Multiple sequence alignment of CscC proteins. Only the last 200 residues are shown. Proteins IDs can be found in additional file Table 5. Colour coding of highly conserved residues: green, hydrophobic (L, M, I, V); blue, aromatic (W, F. Y); red, basic (R, K, H); light brown, neutral and small (P, G, S, T).
