# Supplementary material for: Lactobacillus plantarum gene clusters encoding putative cell-surface protein complexes for carbohydrate utilization are conserved in specific gram-positive bacteria
Source: BMC Genomics. 2006 May 24;7:126. doi: 10.1186/1471-2164-7-126 (PMC1534035; doi:10.1186/1471-2164-7-126)
Supplement: Additional file 9 — Figure 7: Multiple sequence alignment of ConA-like lectins/glucanases domains of CscC proteins. [file 1471-2164-7-126-S9.pdf]

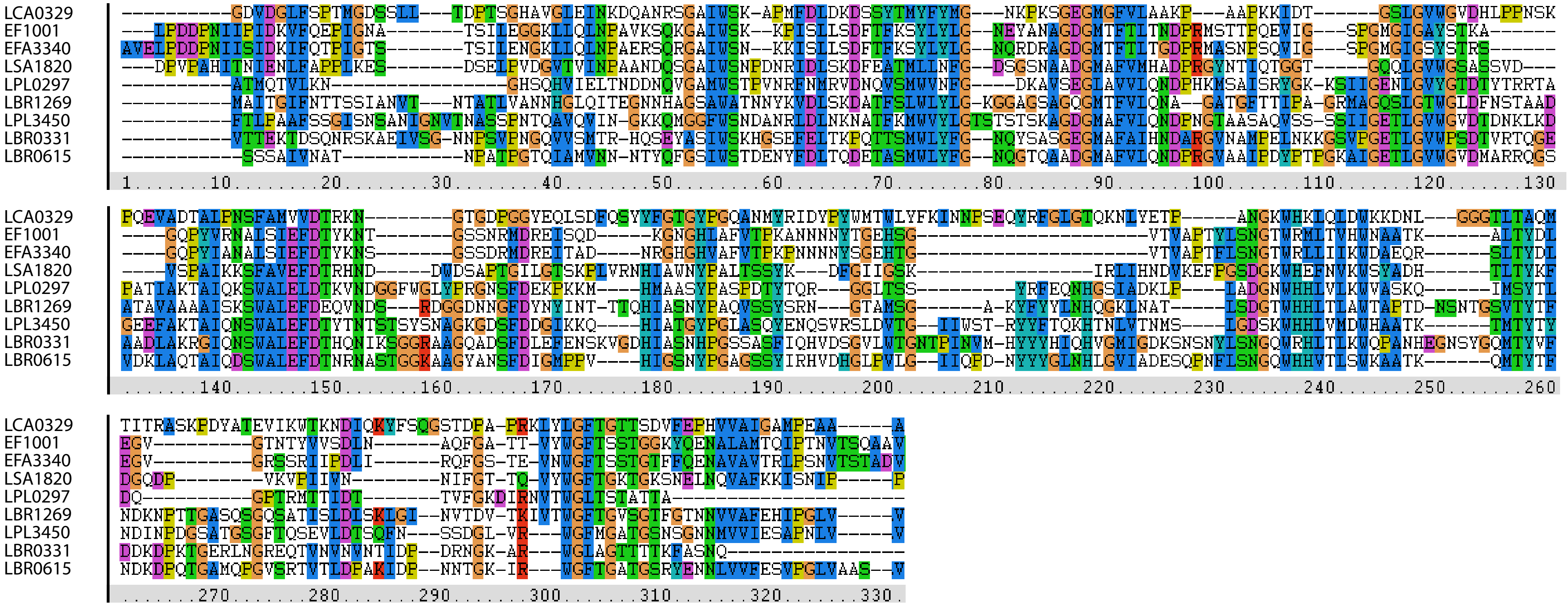

Figure 7:

Multiple sequence aligment of ConA-like lectins/glucanases domains of Csc proteins. See additional file Table 7 for details of domain architectures. Colour coding of highly conserved residues: dark blue, hydrophobic (W, F, L, V, I, M, A); red, basic (R, K); purple, acidic (D, E); green, neutral and small (T, S, N, Q); light blue, cyclic and neutral (Y, H); light brown, glycine (G); olive, proline (P).
