# Supplementary material for: Lactobacillus plantarum gene clusters encoding putative cell-surface protein complexes for carbohydrate utilization are conserved in specific gram-positive bacteria
Source: BMC Genomics. 2006 May 24;7:126. doi: 10.1186/1471-2164-7-126 (PMC1534035; doi:10.1186/1471-2164-7-126)
Supplement: Additional file 12 — Figure 10: Family tree of CscC proteins. [file 1471-2164-7-126-S12.pdf]

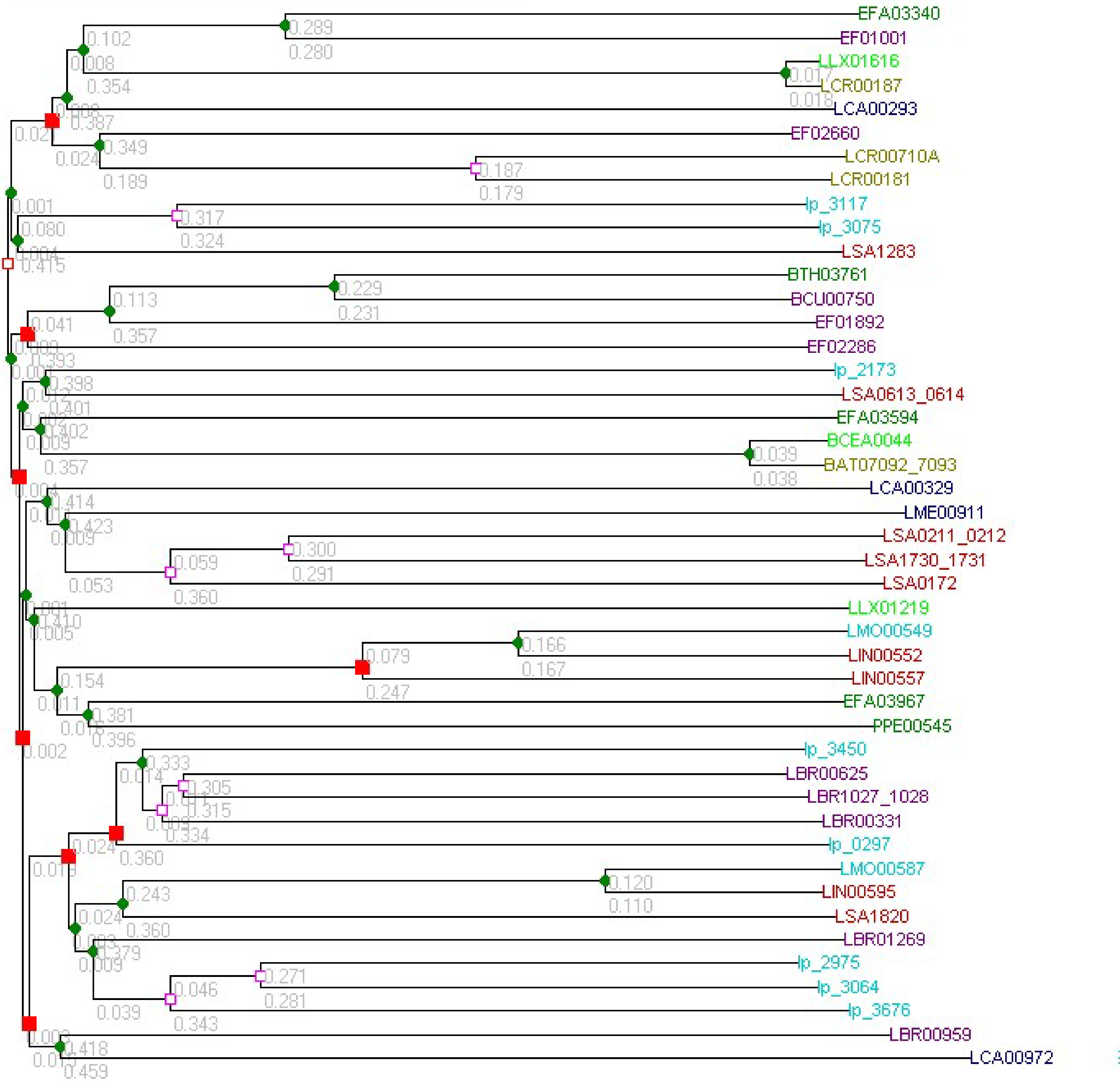

Figure 10:
Protein family tree of CscC proteins, based on alignment in Figure 6 (additional file). Proteins from the same species have the same colour coding. Green dots represent speciation events, and squares represent duplication events (see [1] for details of method).

1. van der Heijden RTJM, Snel B, Huynen MA: LOFT: High resolution multi-level orthology prediction through automated analysis of phylogenetic trees. In: submitted. 2005.
