# Supplementary material for: Lactobacillus plantarum gene clusters encoding putative cell-surface protein complexes for carbohydrate utilization are conserved in specific gram-positive bacteria
Source: BMC Genomics. 2006 May 24;7:126. doi: 10.1186/1471-2164-7-126 (PMC1534035; doi:10.1186/1471-2164-7-126)
Supplement: Additional file 13 — Figure 11: Multiple sequence alignment of ConA-like lectins/glucanases domains of CscC proteins with known 3D structures of lectins. [file 1471-2164-7-126-S13.pdf]

Figure 11: Multiple sequence alignment of ConA-like lectin/glucanase domains with known 3D structures of lectins. Putative ConA-like domains (amino acid numbering in brackets) of representative CscC proteins of *Enterococcus faecalis* (EF), *Enterococcus faecium* (EFA), *Lactobacillus plantarum* (LPL) and *Lactobacillus brevis* (LBR). Known 3D structures with PDB codes: 1hql, lectin, Glechoma hederacea (Ground-ivy); 1n47, lectin B4, Vicia villosa (Hairy vetch); 1qnw, anti-H(O)lectin 2, Ulex europeus (Furze); 1bqp, lectin(precursor), Pisum sativum (Garden pea). The coloured residues represent conserved ligands of divalent metal ions (Mn^{2+} or Mg^{2+}).

```
1hql                  --IRLTTSHT---WSVGRVMYSKPLTFWG--EGKQVHFKTKISFNITSIAGNKA-DGVAL
1n47                  --LELTKNGKPVPESLGRN------------CTTLASFTTSFSFVMSAPNSLDVADGLAF
1qnw                  --LQVTKVSKPTTTSIGRALYAAPIQIWDSITGKVASFATSFSFVVKADKSDGV-DGLAF
2bqp                  --LQLTKAVK---NSIGRALYSAPIHIWDSKTGDVANFETLFTFAITAPYSSNVADGLAF
EFA03340(125-335)     --LQLNPAER---SQRG-AIWSNK--KIS--LLSDFTFKSYLYLG--NQRDRAG-DGMTF
EF01001(150-359)      --LQLNPAVK---SQKG-AIWSKK--PIS--LLSDFTFKSYLYLG--NEYANAG-DGMTF
LPL0297(66-301)       HVIELTNDD----NQVG-AMWSTPVNRFN--MRVDNQVSMWVNFG----DKAVS-EGLAV
LPL3450(75-333)       QAVQVINGK----KQMG-GFWSNDANRLD--LNKNATFKMWVYLGTSTSTSKAG-DGMAF
LBR01269(64-320)      HGLQITEGN----NHAG-SAWATNNYKVD--LSKDATFSLWLYLG-KGGAGSAG-QGMTF
LBR00625(76-335)      ---MVNNNT----YQFG-SIWSTDENYFD--LTQDETASMWLYFG--NQGTQAA-DGMAF
LBR00331(73-344)      ---SMTRHQ----SEYA-SIWSKHGSEFE--LTKPQTTSMWLYFG--NQYSASG-EGMAF
                          :           .                        . :           :*: .

1hql                  FM--------VPVGPPIPNGGAGGNLGLFDSSGVGKS-------------IFAVKFDTH-
1n47                  FL--------APPDTQP--QKRGGFLGLFKDRKHDIS-----------YQSVAVEFDTY-
1qnw                  FL--------APANSQIPSGSSAGMFGLFSSSDSKSS-----------NQIIAVEFDTYF
2bqp                  FI--------APVDTQPQNIGRAGFLGVFNSETYNKS-----------IQTVAVEIDTF-
EFA03340(125-335)     TLTGDPRMASNPSQVI---GSPGMGIGSYSTRSGQPY----------IANALSIEFDTYK
EF01001(150-359)      TLTNDPRMSTTPQEVI---GSPGMGIGAYSTKAGQPY----------VRNALSIEFDTYK
LPL0297(66-301)       VLQNDPHKMSAISRYGK-KSIIGENLGVYGTDTYTRRTAPATIAKTAIQKSWALELDTKV
LPL3450(75-333)       VLQNDPNGTAASAQVSS-SSIIGETLGVWGVDTDNKLKDGEEFAKTAIQNSWALEFDTYT
LBR01269(64-320)      VLQNAG---ATGFTTIPAGRMAGQSLGTWGLDFNSTAADATAVAAAAISKSWALEFDEQV
LBR00625(76-335)      VLQNDPRGVAAIPDYPTPGKAIGETLGVWGVDMARRQGSVDKLAQTAIQDSWALEFDTNR
LBR00331(73-344)      AIHNDARGVNAMPELNKKGSVPGETLGVWPSDTVRTQGEAADLAKRGIQNSWALEFDTHQ
                       :                       :* :                       :: .*

1hql                  -----------ANVWDPPCR------HIGINVNSRVPVAHKCMDDSVNWEDVTL-----S
1n47                  -----------SNVWDPNTT------HIGIDTNTIESKKITP------------------
1qnw                  G--------KAYNPWDPDFK------HIGIDVNSIKSIKTVK---W-DWRNGEVADVVIT
2bqp                  -----------HNTWDPKINR-----HIGINVNCIKSISTTS---WVLENGREANVLV-R
EFA03340(125-335)     NS-------GSSDRMDREITAD----NRGHGHVAFVTPKPNN------------------
EF01001(150-359)      NT-------GSSNRMDREISQ-----DKGNGHLAFVTPKANN------------------
LPL0297(66-301)       NDGGFWGLYPRGNSFDEKPKKM----HMAASYPASPDTYTQR------------GGLTSS
LPL3450(75-333)       NTSTSYSNAGKGDSFDDGIKKQ----HIATGYPGLASQYENQ---SVRSLDVTGIIWSTR
LBR01269(64-320)      NDSRDG---GDNNGFDYNYINTTTQ-HIASNYPAQVSSYSRN---GTAMSGA-------K
LBR00625(76-335)      NASTGGKAAGYANSFDIGMPPV----HIGSNYPGAGSSYIRHVDHGLPVLGIIQPD---N
LBR00331(73-344)      NIKSGGRAAGQADSFDLEFENSKVGDHIASNHPGSSASFIQHVDSGVLWTGNTPINVM-H
                                  :  *            . .

1hql                  INYDEADKIITVRAQ----VGLTKHYDLSHKLDLSTI-----------------------
1n47                  --FDMVYGEKILFAS--------------LVFPVSQD-----------------------
1qnw                  YRAPTKSLTVCLSYP-----SDGTSNIITASVDLKAI-----------------------
2bqp                  FDAHTNVLSVVLSYP-----GLPDSYILSDVVPLKDI-----------------------
EFA03340(125-335)     NNYSGEHTGVTVAPT---FLSNGTWRLLIIKWDAEQR------SLTYDLEGV--------
EF01001(150-359)      NNYTGEHSGVTVAPT---YLSNGTWRMLTVHWNAATK------ALTYDLEGV--------
LPL0297(66-301)       YRFEQNHGSIADKLP----LADGNWHHLVLKWVASKQ------IMSYTLDQ---------
LPL3450(75-333)       YYFTQKHTNLVTNMS----LGDSKWHHLVMDWHAATK------TMTYTYNDINPDGSATG
LBR01269(64-320)      YFYYLNHQGKLNATL-----SDGTWHHLTLAWTAPTD-NSNTGSVTYTFNDKNPTTGASQ
LBR00625(76-335)      YYYGLNHLGVIADESQPNFLSNGQWHHVTLSWKAATK------QMTYTFNDKDPQTGAMQ
LBR00331(73-344)      YYYHIQHVGMIGDKSNSNYLSNGQWRHLTLKWQPANHEGNSYGQMTYVFDDKDPKTGERL
```

```
1hql                   ----------------------LEKKVQVGLSASTG---TNIALHDINYWEF
1n47                   ---------------------ILPEYVRVGFSATTGLNEGVVETH-------
1qnw                   ----------------------LPEWVSVGFSGGVGNA-AEFETHDVLSWYF
2bqp                   ----------------------VPEWVRIGFSAATG—--AEFAEHDIRYWSF
EFA03340(125-335)      -GRSSRIIPDLIRQFG-------STEVNWGFTSSTGTFFQEN-AVAVTRLPS
EF01001(150-359)       -GTNTYVVSDLNAQFG-------ATTVYWGFTSSTGGKYQEN-ALAMTQIPT
LPL0297(66-301)        -GPTRMTTIDTTVFGK------DIRNVTWGLTSTATTANART-LVVFDRLPQ
LPL3450(75-333)        SGFTQSEVLDTSQFNS------SDGLVRWGFMGATGSNSGNN-MVVIESAPN
LBR01269(64-320)       SGQSATISLDLSKLGI--NVTDVTKIVTWGFTGVSGTFGTNN-VVAFEHIPG
LBR00625(76-335)       PGVSRTVTLDPAKIDP-----NNTGKIRWGFTGATGSRYENN-LVVFESVPG
LBR00331(73-344)       NGREQTVNVNVNTIDP-----DRNGKARWGLAGTTTTKFASNQLVVFEKAP-
                                                   *:
```

1hql.pdb: Lectin, Glechoma hederacea (Ground-ivy)
1n47.pdb: Lectin B4, Vicia villosa (Hairy vetch)
1qnw.pdb: Anti-H(O)lectin 2, Ulex europeus (Furze)
1bqp.pdb: Lectin(precursor), Pisum sativum (Garden pea)
