# Supplementary material for: Lactobacillus plantarum gene clusters encoding putative cell-surface protein complexes for carbohydrate utilization are conserved in specific gram-positive bacteria
Source: BMC Genomics. 2006 May 24;7:126. doi: 10.1186/1471-2164-7-126 (PMC1534035; doi:10.1186/1471-2164-7-126)
Supplement: Additional file 14 — Legends to additional Figures. [file 1471-2164-7-126-S14.doc]

**Legends to additional Figures**

**Figure 4:**

Multiple sequence alignment of CscA proteins. Proteins IDs can be found in additional file Table 5. Colour coding of highly conserved residues: green, hydrophobic (L, M, I, V); blue, aromatic (W, F. Y); red, basic (R, K, H); light brown, neutral and small (P, G, S, T).

**Figure 5:**

Multiple sequence alignment of CscB proteins. Proteins IDs can be found in additional file Table 5. Colour coding of highly conserved residues: green, hydrophobic (L, M, I, V); blue, aromatic (W, F. Y); red, basic (R, K, H); light brown, neutral and small (P, G, S, T).

**Figure 6:**

Multiple sequence alignment of CscC proteins. Only the last 200 residues are shown. Proteins IDs can be found in additional file Table 5. Colour coding of highly conserved residues: green, hydrophobic (L, M, I, V); blue, aromatic (W, F. Y); red, basic (R, K, H); light brown, neutral and small (P, G, S, T).

**Figure 7:**

Multiple sequence aligment of ConA-like lectins/glucanases domains of Csc proteins. See additional file Table 7 for details of domain architectures. Colour coding of highly conserved residues: dark blue, hydrophobic (W, F, L, V, I, M, A); red, basic (R, K); purple, acidic (D, E); green, neutral and small (T, S, N, Q); light blue, cyclic and neutral (Y, H); light brown, glycine (G); olive, proline (P).

**Figure 8:**

Protein family tree of CscA proteins, based on alignment in Figure 4 (additional file). Proteins from the same species have the same colour coding. Green dots represent speciation events, and squares represent duplication events (see [1] for details of method).

1. van der Heijden RTJM, Snel B, Huynen MA**: LOFT: High resolution multi-level orthology prediction through automated analysis of phylogenetic tre**es. In*: submitte*d. 2005.

**Figure 9:**

Protein family tree of CscB proteins, based on alignment in Figure 5 (additional file). Proteins from the same species have the same colour coding. Green dots represent speciation events, and squares represent duplication events (see [1] for details of method).

1. van der Heijden RTJM, Snel B, Huynen MA**: LOFT: High resolution multi-level orthology prediction through automated analysis of phylogenetic tre**es. In*: submitte*d. 2005.

**Figure 10:**

Protein family tree of CscC proteins, based on alignment in Figure 6 (additional file). Proteins from the same species have the same colour coding. Green dots represent speciation events, and squares represent duplication events (see [1] for details of method).

1. van der Heijden RTJM, Snel B, Huynen MA**: LOFT: High resolution multi-level orthology prediction through automated analysis of phylogenetic tre**es. In*: submitte*d. 2005.

**Figure 11:**

Multiple sequence alignment of ConA-like lectin/glucanase domains of CscC proteins with lectins of known 3D structure. Putative ConA-like domains (amino acid numbering in brackets) of representative CscC proteins of *Enterococcus faecalis* (EF), *Enterococcus faecium* (EFA), *Lactobacillus plantarum* (LPL) and *Lactobacillus brevis* (LBR). Known 3D structures with PDB codes: 1hql, lectin, Glechoma hederacea (Ground-ivy); 1n47, lectin B4, Vicia villosa (Hairy vetch); 1qnw, anti-H(O)lectin 2, Ulex europeus (Furze); 1bqp, lectin(precursor), Pisum sativum (Garden pea). The coloured residues represent conserved ligands of divalent metal ions (Mn^2+^ or Mg^2+^).

1. van der Heijden RTJM, Snel B, Huynen MA**: LOFT: High resolution multi-level orthology prediction through automated analysis of phylogenetic tre**es. In*: submitte*d. 2005.
